# Network Alterations in Comorbid Chronic Pain and Opioid Addiction: An Exploratory Approach

**DOI:** 10.3389/fnhum.2019.00174

**Published:** 2019-05-29

**Authors:** Rachel F. Smallwood, Larry R. Price, Jenna L. Campbell, Amy S. Garrett, Sebastian W. Atalla, Todd B. Monroe, Semra A. Aytur, Jennifer S. Potter, Donald A. Robin

**Affiliations:** ^1^National Institute of Neurological Disorders and Stroke, National Institutes of Health, Bethesda, MD, United States; ^2^Metholology, Measurement and Statistical Analysis, Texas State University, San Marcos, TX, United States; ^3^Department of Communication Sciences and Disorders, University of New Hampshire, Durham, NH, United States; ^4^Department of Psychiatry, University of Texas Health Science Center San Antonio, San Antonio, TX, United States; ^5^College of Nursing, The Ohio State University, Columbus, OH, United States; ^6^Department of Health Management and Policy, University of New Hampshire, Durham, NH, United States; ^7^Interdisciplinary Program in Neuroscience and Behavior, University of New Hampshire, Durham, NH, United States

**Keywords:** chronic low back pain, opioid addiction, fMRI, pain induction, unified structural equation modeling, vector autoregressive modeling, automated search strategy

## Abstract

The comorbidity of chronic pain and opioid addiction is a serious problem that has been growing with the practice of prescribing opioids for chronic pain. Neuroimaging research has shown that chronic pain and opioid dependence both affect brain structure and function, but this is the first study to evaluate the neurophysiological alterations in patients with comorbid chronic pain and addiction. Eighteen participants with chronic low back pain and opioid addiction were compared with eighteen age- and sex-matched healthy individuals in a pain-induction fMRI task. Unified structural equation modeling (SEM) with Lagrange multiplier (LM) testing yielded a network model of pain processing for patient and control groups based on 19 *a priori* defined regions. Tests of differences between groups on specific regression parameters were determined on a path-by-path basis using *z*-tests corrected for the number of comparisons. Patients with the chronic pain and addiction comorbidity had increased connection strengths; many of these connections were interhemispheric and spanned regions involved in sensory, affective, and cognitive processes. The affected regions included those that are commonly altered in chronic pain or addiction alone, indicating that this comorbidity manifests with neurological symptoms of both disorders. Understanding the neural mechanisms involved in the comorbidity is crucial to finding a comprehensive treatment, rather than treating the symptoms individually.

## Introduction

There is a high prevalence of comorbid chronic pain and opioid addiction, presenting a serious healthcare challenge that has become an epidemic in the United States (Rosenblum et al., 2003; Clark et al., 2008; Barry et al., 2013; Salsitz, 2016). Independently, chronic pain and opioid addiction are difficult to treat, and the comorbidity only increases the difficulty with diagnosis and treatment of the disorders. Patients with a substance use disorder (SUD) and co-occurring physical pain have increased cravings (Tsui et al., 2016) and are more likely to misuse opioids than SUD patients without pain (Potter et al., 2008; Dennis et al., 2015). Impulsive tendencies in chronic pain patients indicate a high risk of illicit opioid use (Vest et al., 2016). As well, opioid use is anticorrelated with pain acceptance and lower pain acceptance rates were associated with higher opioid use rates, but pain intensity had no relationship with opioid use (Lin et al., 2015). Chronic pain is positively associated with substance use disorder severity, psychiatric disorders, psychological distress, medical comorbidities, general physical health problems, medical care utilization, and poorer psychosocial function (Jamison et al., 2000; Rosenblum et al., 2003; Potter et al., 2004; Trafton et al., 2004; Arnow et al., 2006; Tunks et al., 2008; Dominick et al., 2012; Burke et al., 2015; Howe et al., 2015). These comorbid factors are associated with relapse into substance use (Potter et al., 2010) and poor treatment outcomes.

The above challenges are compounded by the fact that opioids are often prescribed as treatment for chronic pain conditions. The effect sizes for opioid treatments are negligible, the associated risks, especially for those of dependency, are high (Ballantyne and LaForge, 2007; Noble et al., 2010). Additionally, chronic opioid use can result in opioid-induced hyperalgesia, increasing pain sensitivity (Lee et al., 2011; Stoicea et al., 2015). Thus, there is a great need for further research addressing the comorbidity of chronic pain (Noble et al., 2010; Chou et al., 2015; Dowell et al., 2016; Volkow and McLellan, 2016). The Centers for Disease Control and Prevention recently released a report providing a set of guidelines for clinicians on prescribing opioids for chronic pain, and the first guideline states that non-pharmacologic and non-opioid pharmacologic treatments should be considered before opioids. If opioids are prescribed, it should be at the lowest effective dose for the shortest duration, and non-pharamcologic therapies, such as mindfulness-based or behavioral therapy approaches, and follow-up monitoring should be used in conjunction (Dowell et al., 2016).

In regard to the brain, pain sensation is not only a peripheral physical phenomenon. Acute pain sensation induces widespread activation spanning regions including the anterior cingulate cortex (ACC), insula, somatosensory cortices, thalamus, basal ganglia, and prefrontal cortices (Tracey, 2005; Chen et al., 2008; May, 2008; Schweinhardt and Bushnell, 2010; Davis and Moayedi, 2013; Schmidt-Wilcke, 2015; Jensen et al., 2016; Morton et al., 2016). Chronic pain disorders often manifest altered processing in and interactions between many of those regions during pain tasks and at rest (Apkarian et al., 2001; Gracely et al., 2002; Moisset and Bouhassira, 2007; Napadow et al., 2010; Baliki et al., 2011; Cifre et al., 2012; Davis and Moayedi, 2013; Schmidt-Wilcke, 2015; Jensen et al., 2016; Martucci and Mackey, 2016; Morton et al., 2016), and chronic pain patients exhibit activation in pain-related structures at lower stimulation levels than healthy controls (Gracely et al., 2002; Giesecke et al., 2004). A recent meta-analysis showed healthy individuals have increased activation likelihood due to painful stimulation in the ACC, insula, and thalamus than chronic pain (Jensen et al., 2016). In addition, gray matter volume and cortical thickness are also decreased in many of the same regions, primarily the ACC, thalamus, basal ganglia, insula, and dorsolateral prefrontal cortex (DLPFC) (Apkarian et al., 2001; May, 2008, 2011; Schmidt-Wilcke, 2008; Davis and Moayedi, 2013; Ivo et al., 2013; Smallwood et al., 2013; Alshuft et al., 2016; Yang et al., 2017). In individuals with CLBP, 1 month of oral morphine consumption resulted in gray matter increases and decreases in pain and reward-related structures (Lin et al., 2016).

Opioid dependence and addiction also affect brain structure and function. Differences in regional dynamics in drug-cue task fMRI have been observed in the ACC, insula, prefrontal cortices, caudate, thalamus, putamen, hippocampus, and amygdala (Langleben et al., 2008; Yang et al., 2009; Wang et al., 2010, 2011, 2014; Lou et al., 2012; Li et al., 2013; Schmidt et al., 2014, 2015b). These regions and the nucleus accumbens exhibit altered functional connectivity at rest in opioid-dependent subjects and heroin addicts (Ma et al., 2010, 2015; Upadhyay et al., 2010; Schmidt et al., 2015a; Zhang et al., 2015). Structurally, opioid-dependent subjects have significantly less gray matter volume bilaterally in the amygdala and nucleus accumbens (Upadhyay et al., 2010; Seifert et al., 2015; Lin et al., 2016) and in frontal and temporal areas (Qiu et al., 2014; Lin et al., 2016; Wollman et al., 2016) and increased gray matter volume in the cingulate (Lin et al., 2016). Administration of oral morphine to healthy subjects undergoing pain stimulation caused the pain-related activations to have smaller spatial extent (Hansen et al., 2015).

Treatment for these disorders must be driven by principles of neural plasticity. Specifically, positive treatment outcomes are linked to targeting neural structures that support a given function. This is known as the “specificity” principle because it has been shown that neural plasticity must specifically target those brain regions or networks that have changed from their normal state (Kleim and Jones, 2008; Cramer et al., 2011). Hence, extensive knowledge of both healthy and abnormal brain structures involved in pain processing and reward circuitry is necessary. While knowledge of the neural substrates of chronic pain or opioid addiction alone is substantial, there are no data on the comorbid disorders, hampering treatment development. It is likely that pain and SUD comorbidity causes complex and unique effects on neural organization. We hypothesize that the comorbidity will result in similar changes but with larger magnitudes than in either of the two disorders alone, and that these synergistic effects will extend to unique brain regions.

Complex functions are supported by a connected network of brain regions, and understanding the function of each region of the network and network connectivity properties is important in determining the underlying neural substrates of disorders. The comorbidity of pain and SUD along with other usual symptoms (e.g., depression, anxiety, and sleep disturbances) makes the typical approach to imaging analysis (e.g., group analysis of conditional contrasts) difficult to use since each impairment contributes distinct neurophysiological response patterns. Hence, this study uses a connectivity approach to understanding this comorbid disorder. Because this clinical population has not been investigated with neuroimaging until now, in this experiment we used an exploratory approach to connectivity analysis that is ideal given the vast possibilities for regional changes. This approach allows for a large number of regions to be entered into the analyses. Further, exploratory connectivity analyses allow for study of neural networks without the bias of preconceived hypotheses and can drive more detailed analyses that focus on the specific neural systems implicated in a disorder.

In this study, the first aim was to identify an optimal network of brain regions and study their connectivity based on coherence of regional activities for patient and healthy control groups. Given that the brain data on the individual comorbid conditions are not available, we argue that we are justified in using a healthy control group in this first study. Much is known about the neuroscience of chronic pain and addiction independently, however, the comorbid patient population is yet unstudied. To accomplish this, we used a unified structural equation modeling (SEM) approach (Kim et al., 2007) that provides a framework for estimating contemporaneous and temporal or lagged relationships through a multivariate vector autoregressive model in conjunction with an automated Lagrange multiplier (LM) model testing strategy (Gates et al., 2010). Our second aim was to determine if statistical differences in magnitude existed between groups based on regional alterations. The network structure identified in aim one was evaluated to determine if statistical differences existed between patient and control groups for each path specific to the magnitude and sign of the regression weights.

## Materials and Methods

Eighteen (39.2 ± 12.8 years; 10 males) opioid-addicted individuals with chronic low back pain were recruited from methadone clinics in San Antonio, TX. Eighteen healthy (39.5 ± 12.4 years) individuals were recruited as sex and age (within ±3 years) matches to the patients. Patients met the requirements for current DSM-IV opioid dependence, were currently enrolled in opioid replacement therapy (i.e., methadone maintenance or buprenorphine therapy) for more than 30 days, and had been experiencing chronic low back pain for at least 12 months at a level of 5 or greater on a 0 to 10 rating scale. Control participants had no drug use within the past 30 days, had no drug dependence within the past year, rated their pain-related functional interference as less than 2 on a scale from 0 to 10, and considered themselves healthy. This study was carried out in accordance with the recommendations of the University of Texas Health Science Center San Antonio’s Internal Review Board with written informed consent from all subjects. All subjects gave written informed consent in accordance with the Declaration of Helsinki.

Participants completed a battery of paper questionnaires to assess pain and addiction severity, including the Acceptance and Action Questionnaire II (AAQ-II; Bond et al., 2011), the Mindfulness Attention Awareness Scale (MAAS; Brown and Ryan, 2003), the Roland Morris Disability Questionnaire (RMDQ; Roland and Morris, 1983), and the visual analog scale for opioid craving, interference, and intensity (VAS; McMillan and Gilmore-Thomas, 1996). All demographic and assessment data have been included in Table 1. While outside of the scanner, participants also underwent a threshold test to determine their individualized pain stimulation levels. Pain stimuli were delivered via pressure to the right thumbnail with a pneumatic device.

**Table 1:** Participant demographics and assessment results.

	HC	CPOA matched
Subjects (N)	18	18
Gender	10 males, 8 females	10 males, 8 females
Age (years)	39.5 ± 12.4	39.2 ± 12.8
Acceptance and action questionnaire II (AAQ-II)	62.0 ± 9.0^∗^	39.2 ± 9.0
Mindfulness Attention Awareness Scale (MAAS)	4.8 ± 0.9^∗^	3.6 ± 0.9
Visual analog scale (VAS), opioid craving	0.0 ± 0.0	2.4 ± 2.6
Visual analog scale (VAS), interference	0.0 ± 0.0^∗^	3.7 ± 2.0
Visual analog scale (VAS), intensity	0.0 ± 0.0^∗^	3.8 ± 2.1
Roland Morris Disability Questionnaire (RMDQ, %)	–	53.0 ± 24.5


*Averages and standard deviations listed; HC, healthy controls; ^∗^healthy controls significantly different from matched patients, *p* ≤ 0.001.*

Patients underwent a 16-min pain induction fMRI task containing 8 triplets of 5-s painful pressure blocks (pressure the subjects rated as 40/100 on a pain scale) and 8 triplets of 5-s innocuous pressure blocks (pressure that was not rated as painful), each followed by rest periods. An anatomical scan for registration was also collected. Data were collected using a 3T Siemens TIM Trio scanner (fMRI TR/TE/tip angle/slices/voxel size = 2500 ms/30 ms/90°/36/1.72 × 1.72 × 3 mm; aMRI TR/TE/TI/flip angle/voxel size = 2200 ms/2.8 ms/766 ms/13°/1 × 1 × 1 mm). The pain task fMRI data were pre-processed and analyzed using SPM8. To begin, motion parameters were observed across the entire time series of the scan. If a subject had a large spike in motion (≥1 mm/TR), the ArtRepair toolbox was used to interpolate signal from the preceding and following volumes. Then either the raw data (if no motion correction was needed) or the artifact-repaired data were realigned and resliced, coregistered to the anatomical image, transformed into MNI standard space using the transformation derived from the segmented anatomical image, and then smoothed with an 8 mm FWHM kernel. The functional time series for each volume of interest (VOI) was extracted, normalized, and adjusted for motion. Each VOI was centered on the coordinate specified in Table 2 and was spherical with a 6 mm radius. The 19 regions subjected to analyses are included in Table 2. The effective sample size was *N* = 1153 in the control group (pain condition) and *N* = 1153 in the patient group (pain condition). The effective sample size was *N* = 6912 for the control group under all experimental conditions (pain + innocuous + rest) and *N* = 6903 for the patient group under all experimental conditions.

**Table 2:** Volumes of interest included in model 2.

Region	*X*	*Y*	*Z*	Abbreviation
Left insula	–40	6	2	lIns
Right insula	41	15	1	rIns
Dorsal anterior cingulate cortex	3	36	22	dACC
Left amygdala	–23	–3	–17	lAmyg
Right amygdala	23	–4	–16	rAmyg
Left dorsolateral prefrontal cortex	–31	43	22	lDLPFC
Right dorsolateral prefrontal cortex	41	39	24	rDLPFC
Left putamen	–25	0	5	lPut
Right putamen	25	7	2	rPut
Left caudate	–12	4	13	lCaud
Right caudate	15	9	14	rCaud
Left thalamus	–13	–11	16	lThal
Right thalamus	9	–11	7	rThal
Left primary somatosensory cortex	–57	–24	23	lS1
Right primary somatosensory cortex	58	–24	21	rS1
Left precuneus	–18	–57	34	lPrecun
Right precuneus	19	–57	35	rPrecun
Left nucleus accumbens	–9	6	–4	lNAcc
Right nucleus accumbens	9	6	–4	rNAcc


*Center coordinates in MNI space of 6 mm radius spheres provided.*

## Analytic Strategy

In functional connectivity studies, the goal includes modeling the temporal effect of neural activation in one region in relation to another region. However, each observation (single fMRI volume) is partly a function of the previous within-subject observation due to the multiple volumes collected for each subject. The interdependence among the observations within subjects is manifested in the within-subject residual error of regression for one observation at time *t* (contemporaneous component) correlating with the previous measurement at time *t*-1 (temporal component). The autoregressive effect is typically positive thereby biasing the standard errors of regression estimates downward, yielding *F*-statistics with inflated statistical significance (Bingenheimer and Raudenbush, 2004). Kim et al. (2007) provided a unified SEM approach that allows for estimation of contemporaneous relations (e.g., at time *t*) among ROIs controlling for sequential dependencies present in fMRI data structures. The unified SEM approach also provides a framework for estimating vector autoregressive parameters (i.e., lagged relationships – time *t*-1) after controlling for contemporaneous effects. For example, interest may lie in the effect of region *X* at time *t*-1 on region *Y* at time *t* (current time). This autoregressive analytic approach is then expanded throughout the multivariate regression (network) model to estimate the path loadings throughout the network (Kim et al., 2007; Price, 2012).

Thus, the unified SEM approach advances current techniques by providing a flexible, dynamic approach for simultaneously estimating contemporaneous and lagged relationships between ROIs. Although Granger Causality Modeling can be used to estimate lagged relationships, biased estimates may result from failing to consider contemporaneous relations (Gates et al., 2010). Dynamic causal modeling (DCM) is another approach that can be used to study event-related data. However, DCM is limited to modeling contemporaneous change, whereas the unified SEM is appropriate for simultaneously modeling contemporaneous and lagged effects. Additionally, DCM is used for confirmatory analysis, while the unified SEM approach is appropriate for either confirmatory or exploratory analysis. Because it is entirely data-driven, the unified SEM offers a substantial degree of flexibility when compared to alternative approaches (Gates et al., 2010, 2011; Guàrdia-Olmos et al., 2018).

### Identifying the Network Structure

Prior to analyses, we conducted data screening to evaluate the time series properties of the data. Data screening included evaluating (a) the stationarity or non-stationarity of the time series, (b) the time period between observations to determine the lag structure (e.g., lag-1, lag-2, or lag-3) and (c) the autocorrelation and partial autocorrelation functions. Results of the data screening (i.e., autocorrelation and partial autocorrelation plots of residuals) revealed a stationary, white noise process with a lag-1, the time series best representing the series (Box et al., 2015).

The present study was exploratory given the lack of previous research on the comorbidity of chronic pain and opioid addiction. Therefore, although the regions of interest were selected *a* *priori*, no network model of functional connectivity between those regions was posited *a priori*. Consequently, we employed a search strategy involving LM testing with forward selection starting with a null model (no regression paths among regions) then sequentially added additional parameters one step at a time (Chou and Bentler, 1990; Gates et al., 2010). This process continued until the first non-significant path loading was observed. This search algorithm was conducted using Linear Structural Relations (LISREL), version 9.2 (Jöreskog and Sörbom, 2015). Table 3 provides the fit statistics for the final model for the patient and control groups. Supplementary Tables S1, S2 show all the connections present in the optimal models in all subjects for all conditions and the pain condition, respectively. To compare connections between groups, a *Z*-test was employed on the Fisher’s Z for each connection for each group.

**Table 3:** Summary fit statistics pain condition, all conditions.

	Control	Patients	Controls	Patients
*X*^2^	2520.64	4594.89	11341.75	23431.75
*df*	590	590	590	590
*p*	<0.001	<0.001	<0.001	<0.001
CFI	0.93	0.90	0.92	0.92
RMSEA	0.05	0.07	0.05	0.07
Stability Index	0.46	0.51	0.53	0.53


*X^2^-test of overall model fit; *df*, degrees of freedom; *p*, probability level; CF, comparative fit index; RMSEA, root mean square error of approximation; Stability Index is a measure of system stability in a non-recursive structural equation model (Fox, 1980; Bentler and Freeman, 1983). Pain condition, left; all conditions, right.*

## Results

Almost every connection in the model was significantly different between groups because there was such a large sample size; therefore, an effect size (Cohen’s q; Cohen, 1988) was calculated to distinguish the most relevant and meaningful differences. The results and discussion will focus on the connections that were significantly different between patients and controls with at least a moderate effect size (*q* ≥ 0.3). The between group differences for all connections (regardless of effect size) can be seen in Supplementary Tables S1, S2. All of the significantly different connection strengths with large or moderate effect sizes were greater in patients than in control subjects. Although there were some connection strengths that were greater in controls than patients, as indicated by a negative z-score, effect sizes for these comparisons were small (*q* ≤ 0.21 for all conditions, *q* ≤ 0.19 for pain condition only).

**FIGURE 1:**
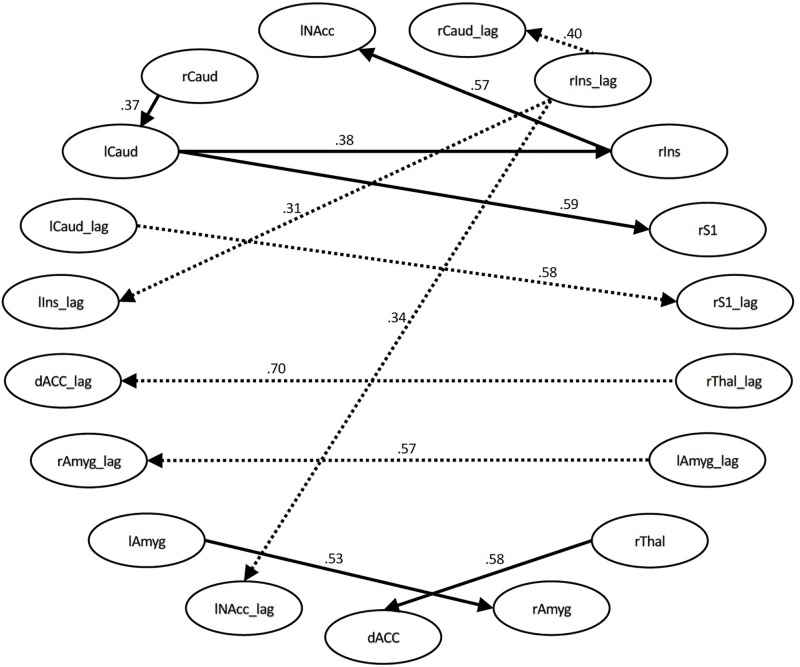
Structural equation modeling network model – all. Patient and Control Groups, All Conditions. Numbers on paths are effect sizes representing the difference between Controls and Patients under all conditions.

**FIGURE 2:**
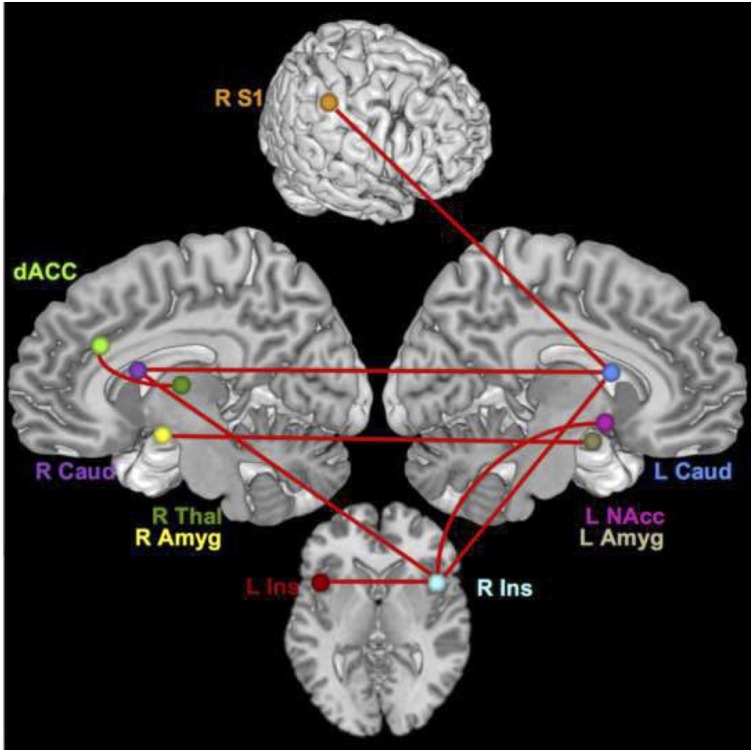
Brain network regions – all. Connections that differed significantly between groups with a moderate or large effect size for all conditions.

For the time series with all conditions included, the connections that differed significantly with large or moderate effect sizes were the connection between the right thalamus and dACC (effect size *q* = 0.58, lag *q* = 0.70), the right S1 and left caudate (*q* = 0.59, lag *q* = 0.58), the right insula and left NAcc (*q* = 0.57, lag *q* = 0.34), the right amygdala to left amygdala (*q* = 0.53, lag *q* = 0.57), the right insula and right caudate (lag *q* = 0.40), the right insula to left caudate (*q* = 0.38), the right caudate and left caudate (*q* = 0.37), and the right insula and left insula (*q* = 0.31). See Figure 1, 2.

**FIGURE 3:**
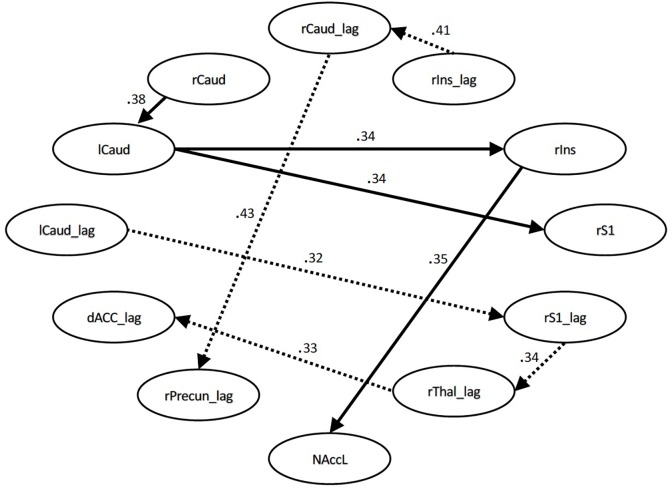
Structural equation modeling network model – pain. Patient and Control Groups, Pain Condition. Numbers on paths are effect sizes representing the difference between Controls and Patients under pain condition only.

**FIGURE 4:**
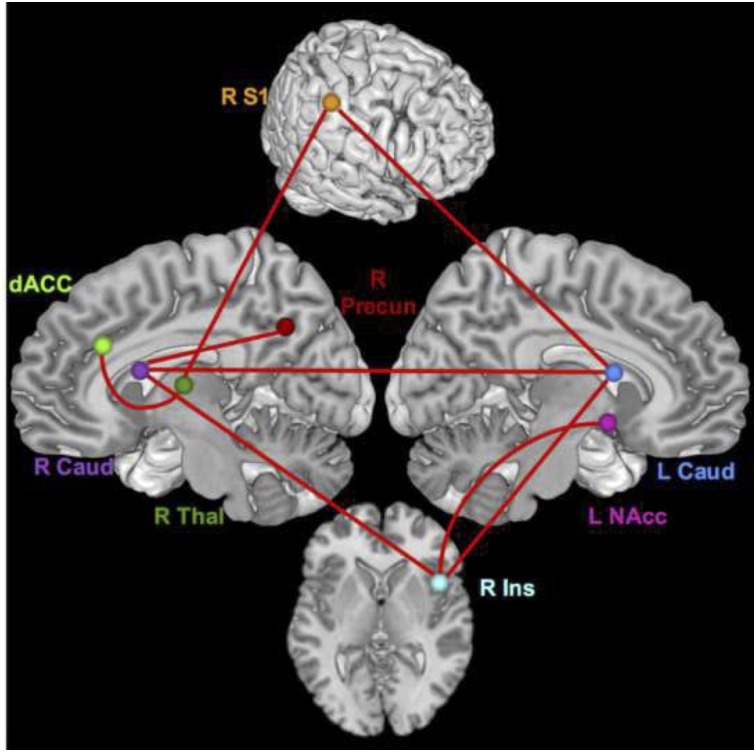
Brain network regions – pain. Connections that differed significantly between groups with a moderate effect size for the pain condition.

During the pain conditions, there were only connection strength differences with moderate effect sizes. These connections were between the right S1 and left caudate (*q* = 0.34, lag *q* = 0.43), the right insula and right caudate (lag *q* = 0.41), the right caudate and left caudate (*q* = 0.38), the right insula and left NAcc (*q* = 0.35), right insula and left caudate (*q* = 0.34), right S1 and right thalamus (lag *q* = 0.34), right thalamus and dACC (lag *q* = 0.33), and the right caudate and right precuneus (lag *q* = 0.32). See Figure 3, 4.

## Discussion

This study is to our knowledge the first to characterize the neural networks underlying comorbid chronic pain and opioid addiction. Our results indicate that the network changes occurring in patients with this comorbidity reflect a combination of the changes observed in chronic pain and addiction alone. Because of the novelty of the population we used an exploratory analysis to determine the network model via SEM using an automated search algorithm that implements LM testing. Group differences were quantified based on this network model. Hence, the critical analysis from these data is the quantification of coupling properties between model regions. Specifically, higher connection values denote stronger connection strengths, with the inference being two regions with similar temporal activity fluctuations are working in concert during the processing of stimuli. The connections that varied between groups, demonstrating a medium or greater effect size (|q|≥ 0.3), reflected *higher connection strengths* in patients compared with controls (positive q). This indicates an increase in coherence of activity between seed regions during painful stimulation in opioid- addicted patients with CLBP compared to healthy subjects.

Previous studies in chronic pain have also reported altered connectivity in patients at rest, showing differences in the default mode network (DMN) (Baliki et al., 2008, 2014; Napadow et al., 2010; Bolwerk et al., 2013; Otti et al., 2013; Kucyi et al., 2014; Hemington et al., 2016; Mansour et al., 2016; Letzen and Robinson, 2017; Yang et al., 2017), executive attention network (Napadow et al., 2010), salience network (Otti et al., 2013; Hemington et al., 2016), in the insula, ACC, and basal ganglia (Malinen et al., 2010; Cifre et al., 2012; Schwedt et al., 2013; Yang et al., 2017), during spontaneous back pain (Hashmi et al., 2013), and during painful stimulation (Baliki et al., 2010; Jensen et al., 2013). Similar connectivity alterations have been observed in opioid-dependent and -addicted populations, with the alterations occurring in amygdala, insula, NAcc, prefrontal cortex (Ma et al., 2010; Upadhyay et al., 2010; Zhang et al., 2011; Schmidt et al., 2014, 2015b), orbitofrontal cortex, caudate, parahippocampus, lingual gyrus, precuneus, middle temporal gyrus (Chang et al., 2016), putamen, posterior cingulate (Schmidt et al., 2015a), and anterior cingulate (Zhang et al., 2015). Our results, coupled with existing literature, indicate both conditions likely contribute to connectivity alterations and that patients’ neurophysiological responses to induced pain are characterized by stronger connections between regions than normal controls’ responses. This increased synchronicity between regions in patients could reflect an increase in communication with more information being passed between the regions; it could also be indicative of an alteration in a common upstream or regulatory region that is passed to its downstream effectors. Whatever the mechanism, these patterns are consistent with central sensitization (Ng et al., 2017); the neuroplastic changes that occur in chronic pain could lead to regions being more functionally connected than necessary for typical pain processing.

The regions with differing connections are not only regions associated with sensory discrimination of pain (S1, thalamus, insula), but also regions that are involved in the emotional response to pain (insula, caudate, amygdala, dACC) and higher-level regulation and integration of pain signals (caudate, nucleus accumbens, dACC) (Tracey, 2005; Chen et al., 2008; May, 2008; Baliki et al., 2010; Schweinhardt and Bushnell, 2010). If individuals with chronic pain and opioid addiction simply had lower pain thresholds, we would expect to see differences primarily, or even exclusively, in sensorimotor regions. The increased coupling strength of sensory regions with others could partially underlie differences from normal. However, the diverse functional nature of regions with stronger connectivity signifies that this population likely has a heightened multi-dimensional response to pain (i.e., sensory, affective, and cognitive), not just in pain sensation. This is in agreement with a finding that chronification of back pain coincides with a shift of processing from more sensory/acute pain circuits to affective circuits (Hashmi et al., 2013), and CLBP patients appear to have more alterations in regions associated with emotion and cognition than in nociceptive regions (Woolf, 2011). Another interesting trend was many of the significant connections with moderate effect sizes were interhemispheric, indicating that although the painful stimulation was only applied on the right thumb, pain processing in this clinical population seems to be characterized by increased bilateral engagement. This is an intriguing consideration in the context of a recent study of normal pain processing that revealed higher pain stimulus levels resulted in increased interhemispheric DLPFC connectivity (Sevel et al., 2016). Perhaps the constant state of pain in the patients causes these plastic changes in bilateral connections.

Additionally, considering the task used in this study highlights an important trend in the regional connectivity. A pain induction paradigm would assume activation and coordination of thalamus and S1 which primarily encode the sensory aspects of pain. However, another important feature to note in the difference network is that, other than thalamus and S1, all of the regions are altered in both diagnoses independently. The observable differences manifesting in regions that overlap between chronic pain and opioid addiction is consistent with the suggestion of chronic pain and addiction following similar neuroadaptation patterns based on a common neural substrate foundation (Elman and Borsook, 2016). It could be that these are regions where the two disorders work synergistically to cause alterations. This finding provides strong support for the development of treatments that simultaneously treat the two disorders, rather than treating one and/or the other independently. Though there have only been a couple of clinical studies with this approach, they have promising results for patients with comorbid chronic pain and opioid addiction (Ilgen et al., 2016; Smallwood et al., 2016).

The observed increases in connectivity likely are not only related to the actual pain stimulus, but evince aberrant connectivity due to addiction. The NAcc is a central part of reward circuitry (Martin-Soelch et al., 2001) and is altered in opioid-dependent subjects (Ma et al., 2010; Upadhyay et al., 2010). It was also predictive of the effect of pain stimuli on chronic pain in a pain induction study in chronic back pain (Baliki et al., 2010). Its role here suggests its participation could be part of a mechanism underlying the emotional response to pain in the form of a trigger for the substance dependence-related response. This is consistent with the between-group difference in its connection with the insula. The insula has a key role in pain processing, being responsible for both sensory and affective aspects of pain (Tracey, 2005; Chen et al., 2008; Schweinhardt and Bushnell, 2010). In painful stimulation of healthy subjects, it was shown that its connectivity shifted with modulation of attention and emotion (Ploner et al., 2011). The insula is also one of the regions commonly activated in tasks when heroin addicts are shown heroin cues in the scanner (Langleben et al., 2008; Lou et al., 2012). Naqvi and Bechara (2010) proposed a drug cue-induced model of processing that includes a connection between the insula and NAcc. They hypothesized that observing a cue previously associated with partaking of a particular addictive substance would activate a network in which the insula acts as a gate to allow previous experiences of the substance’s effects to intensify the urge use, represented by the nucleus accumbens within the reward system. Perhaps in this population painful sensations and their affective sequelae trigger the association of the analgesic effect of opioids, thus increasing the individual’s craving for pain relief and the high experienced from the opioids. This highlights a unique challenge to treating addiction and dependence in patients with comorbid chronic pain: if the presence of the pain creates an additional drive for substance use, these individuals could be fighting an even stronger impulse to use. Furthermore, qualitative research suggests that patients with comorbid chronic pain and SUD perceive that healthcare providers are not treating their pain and addiction in an integrated manner (St. Marie, 2014), thereby generating heightened cravings and perpetuating substance misuse.

The caudate is hypothesized to be responsible for regulating the affective response to pain (Borsook et al., 2010), so the connection between S1 and caudate is likely a pathway for transduction from a sensory-only experience to a multi-dimensional experience that includes affective and higher order cognitive/regulatory components. The increased connectivity in patients between the caudate and the precuneus during the pain only condition could be indicative of an increased affective response to pain in patients due to increased pain sensitivity (Goffaux et al., 2014). The amygdala receives nociceptive inputs from the brain, but also encodes a plethora of affective processes (Veinante et al., 2013) and has been linked with craving-relating activation in response to drug cues in opioid-dependent subjects (Murphy et al., 2017). The bilateral amygdala connection differing significantly could imply an increase in the emotional response to pain, but since the difference was observed only in the time series with all conditions (including rest and innocuous pressure) and not during pain induction alone, perhaps it signifies patients having an increased fearful response or negative anticipation of the coming pain compared with controls.

It is important to underline here that since we are not reporting longitudinal or structural MRI data, we cannot conclude on the neural network and morphological changes that may have occurred in the patient group after withdrawing from opioids. All patients had been enrolled in an opioid replacement therapy program for at least 30 days prior to data collection. Fingelkurts et al. (2009) reported that measures of local and remote electroencephalogram (EEG) functional connectivity of opioid-dependent patients treated with methadone for 6 months did not differ significantly from normal values observed in healthy controls. Studies of medication-overuse headache (MOH), which has also been associated with psychiatric comorbidities, report that in some patients gray matter volume changes reverted to normal state after a period of drug withdrawal. Namely, an increase in gray matter in the orbitofrontal cortex and a decrease in periaqueductal gray region of the midbrain were observed and these changes positively correlated with treatment response (Riederer et al., 2013; Lai et al., 2016). To address this issue, future studies should include both longitudinal and voxel-based morphometry (VBM) data.

Our study provides a novel approach to modeling network structure and connectivity patterns, though we address a few limitations here. First, the population of opioid-addicted individuals with chronic pain was very heterogeneous. Ideal exclusion criteria should include a variety of psychiatric disorders. However, this population included participants with a range of comorbid psychiatric conditions such as depression, anxiety, bipolar disorder, and schizophrenia. Nearly every participant self-reported some type of psychiatric condition, often more than one. This is consistent with data reported in a review by Kelly and Daley (2013), stating that 27% of people with SUD have at least one psychiatric disorder and 45% of people with psychiatric conditions actually have two or more disorders (Kessler et al., 2005). These conditions were self-reported, and had they been excluded there would not have been a large enough population to conduct a study with this comorbidity. Second, another constraint that plagues studies of comorbidities is that it is unknown how two (or more) comorbid disorders interact and whether they interact uniformly in all patients. This introduces the potential for more heterogeneity, and these sources of heterogeneity are one of the primary impetuses for using an exploratory approach. Additionally, any differences observed cannot be ascribed to one diagnosis or the other, as we only have the comorbid patient population and a negative control population. Future studies should have positive control groups including subjects with only chronic low back pain and only opioid addiction. However, we feel strongly that although we cannot specifically attribute any of these differences or characteristics to one diagnosis, the other, or the comorbidity, these results still provide essential knowledge about a pragmatic clinical population (Ford and Norrie, 2016) that represents one of our current significant healthcare challenges.

## Conclusion

The results presented here show that in a network determined via an exploratory SEM analysis, opioid-addicted chronic pain patients had increased connectivity in regions that are affected in both disorders independently. These increases likely indicate altered emotional responses to pain as well as addiction-related neurophysiological reactions, signifying that this comorbidity may act in a synergistic way to exacerbate neural alterations.

This analytic approach represents a novel and interesting way to examine connectivity data. The SEM allowed for defining and refining an optimal network for all subjects. The feature of the SEM that allowed for a large number of regions to be included in the model was invaluable from the exploratory side of the analysis. Because this is a novel population for neuroimaging study, having few restrictions on the number of regions (nodes) of interest included in the model and requiring no *a priori* hypotheses about model structure allowed a broader investigation of the potential relationships and alterations between brain regions in this cohort.

## Ethics Statement

This study was approved and carried out in accordance with the recommendations of the University of Texas Health Science Center San Antonio’s Internal Review Board with written informed consent from all subjects. All subjects gave written informed consent in accordance with the Declaration of Helsinki.

## Author Contributions

RS conducted all the study aspects, development, implementation, interpretation, and wrote the manuscript. LP developed the connectivity models, assisted in model interpretation, and ran all the statistical tests on model connections and wrote the manuscript. JC, SAA, TM, SWA, and AG contributed to proofreading, provided the input into final document, assisted with writing and interpretation of the data, and contributed pertinent supporting references. JP assisted in study design, interpretation of data, and wrote the manuscript. DR supervised and was invovled in all aspects of study development, implementation, analysis, and interpretation.

## Conflict of Interest Statement

The authors declare that the research was conducted in the absence of any commercial or financial relationships that could be construed as a potential conflict of interest.
