# Supplementary material for: Network Alterations in Comorbid Chronic Pain and Opioid Addiction: An Exploratory Approach
Source: Front Hum Neurosci. 2019 May 29;13:174. doi: 10.3389/fnhum.2019.00174 (PMC6548857; doi:10.3389/fnhum.2019.00174)
Supplement: Supplementary file 1 [file Table_1.DOCX]

**Supplementary Table 1.** **Connection statistics all conditions.**

|  |  |  | **Control Group - All Conditions** | | | | | |
| --- | --- | --- | --- | --- | --- | --- | --- | --- |
| Path | | | *B* | *SE B* | C.R. | **β** | Z' | P |
| CaudR_1 | **<---** | InsR_1 | 0.47 | 0.01 | 37.02 | 0.41 | 0.43 | *** |
| CaudL_1 | **<---** | CaudR_1 | 0.84 | 0.01 | 75.92 | 0.65 | 0.78 | *** |
| CaudR | **<---** | CaudR_1 | -0.18 | 0.01 | -14.82 | -0.18 | -0.18 | *** |
| CaudL_1 | **<---** | InsR_1 | 0.30 | 0.01 | 23.70 | 0.20 | 0.21 | *** |
| CaudL | **<---** | CaudR | 0.92 | 0.01 | 89.03 | 0.72 | 0.90 | *** |
| CaudL | **<---** | CaudL_1 | -0.13 | 0.01 | -16.63 | -0.13 | -0.13 | *** |
| S1R | **<---** | CaudL | 0.29 | 0.01 | 26.22 | 0.37 | 0.39 | *** |
| S1R_1 | **<---** | CaudL_1 | 0.29 | 0.01 | 26.22 | 0.37 | 0.39 | *** |
| InsR | **<---** | CaudL | 0.32 | 0.01 | 44.15 | 0.47 | 0.51 | *** |
| S1R_1 | **<---** | CaudR_1 | 0.29 | 0.01 | 19.92 | 0.28 | 0.29 | *** |
| S1R | **<---** | CaudR | 0.29 | 0.01 | 19.90 | 0.28 | 0.29 | *** |
| ThalL | **<---** | InsR | 0.30 | 0.01 | 32.39 | 0.32 | 0.34 | *** |
| ThalR_1 | **<---** | CaudR_1 | 0.52 | 0.02 | 28.33 | 0.39 | 0.42 | *** |
| ThalL | **<---** | InsR_1 | 0.07 | 0.01 | 7.32 | 0.07 | 0.07 | *** |
| ThalL | **<---** | S1R | 0.34 | 0.01 | 42.45 | 0.43 | 0.45 | *** |
| ThalL_1 | **<---** | S1R_1 | 0.34 | 0.01 | 43.02 | 0.43 | 0.46 | *** |
| ThalL_1 | **<---** | InsR_1 | 0.30 | 0.01 | 31.35 | 0.31 | 0.32 | *** |
| ThalR_1 | **<---** | CaudL_1 | 0.28 | 0.01 | 19.67 | 0.27 | 0.28 | *** |
| PutaL | **<---** | ThalL | 0.23 | 0.01 | 21.82 | 0.25 | 0.26 | *** |
| PutaL | **<---** | InsR | 0.18 | 0.01 | 18.63 | 0.22 | 0.22 | *** |
| InsL_1 | **<---** | ThalL_1 | 0.35 | 0.02 | 21.67 | 0.25 | 0.26 | *** |
| InsL_1 | **<---** | ThalR_1 | 0.24 | 0.01 | 25.96 | 0.28 | 0.29 | *** |
| InsL_1 | **<---** | ThalL | 0.02 | 0.01 | 1.67 | 0.02 | 0.02 | 0.095 |
| PutaL | **<---** | CaudR | 0.16 | 0.01 | 19.50 | 0.22 | 0.22 | *** |
| InsL_1 | **<---** | InsR_1 | 0.19 | 0.02 | 12.81 | 0.15 | 0.15 | *** |
| PrecnR_1 | **<---** | PutaL | 0.14 | 0.02 | 8.80 | 0.14 | 0.14 | *** |
| PutaL_1 | **<---** | InsL_1 | 0.10 | 0.01 | 11.23 | 0.16 | 0.16 | *** |
| PrecnR_1 | **<---** | ThalL_1 | 0.41 | 0.02 | 19.73 | 0.46 | 0.50 | *** |
| PrecnR_1 | **<---** | CaudR_1 | 0.16 | 0.02 | 9.57 | 0.23 | 0.23 | *** |
| PrecnR_1 | **<---** | CaudL_1 | 0.07 | 0.01 | 5.26 | 0.12 | 0.12 | *** |
| PrecnR_1 | **<---** | InsL_1 | 0.25 | 0.02 | 14.84 | 0.38 | 0.40 | *** |
| ThalR | **<---** | CaudR | 0.52 | 0.02 | 28.31 | 0.39 | 0.42 | *** |
| ThalR | **<---** | CaudL | 0.28 | 0.01 | 19.67 | 0.27 | 0.28 | *** |
| InsL | **<---** | PutaL | 0.47 | 0.02 | 27.09 | 0.30 | 0.31 | *** |
| dACC_1 | **<---** | InsR_1 | 0.03 | 0.01 | 3.22 | 0.03 | 0.03 | 0.001 |
| InsL | **<---** | ThalR | 0.23 | 0.01 | 25.82 | 0.27 | 0.28 | *** |
| dACC_1 | **<---** | ThalR_1 | 0.39 | 0.01 | 55.80 | 0.56 | 0.63 | *** |
| InsL | **<---** | ThalL | 0.26 | 0.02 | 17.01 | 0.19 | 0.19 | *** |
| dACC_1 | **<---** | InsL_1 | 0.14 | 0.01 | 16.34 | 0.17 | 0.17 | *** |
| S1L | **<---** | InsL | 0.44 | 0.01 | 42.57 | 0.42 | 0.44 | *** |
| S1L | **<---** | CaudR | 0.13 | 0.01 | 9.24 | 0.11 | 0.11 | *** |
| S1L_1 | **<---** | ThalL_1 | 0.16 | 0.02 | 10.89 | 0.11 | 0.11 | *** |
| S1L_1 | **<---** | InsL_1 | 0.43 | 0.01 | 39.34 | 0.41 | 0.43 | *** |
| S1L_1 | **<---** | dACC_1 | 0.34 | 0.01 | 26.54 | 0.27 | 0.27 | *** |
| S1L_1 | **<---** | CaudL_1 | 0.11 | 0.01 | 11.17 | 0.12 | 0.12 | *** |
| S1L | **<---** | S1R | 0.19 | 0.01 | 15.26 | 0.16 | 0.16 | *** |
| S1L | **<---** | ThalR | 0.18 | 0.01 | 17.68 | 0.20 | 0.20 | *** |
| dlpfcR_1 | **<---** | InsL_1 | 0.66 | 0.01 | 67.74 | 0.64 | 0.75 | *** |
| PutaR | **<---** | S1L | 0.41 | 0.01 | 43.70 | 0.48 | 0.52 | *** |
| PutaR | **<---** | InsL | 0.27 | 0.01 | 28.72 | 0.29 | 0.30 | *** |
| PutaR | **<---** | CaudR | 0.06 | 0.01 | 5.27 | 0.06 | 0.06 | *** |
| PutaR | **<---** | ThalR | 0.02 | 0.01 | 2.45 | 0.03 | 0.03 | 0.014 |
| dlpfcR_1 | **<---** | S1L_1 | 0.21 | 0.01 | 22.56 | 0.21 | 0.21 | *** |
| dlpfcR | **<---** | PutaR | 0.31 | 0.01 | 30.24 | 0.28 | 0.28 | *** |
| dlpfcR | **<---** | InsL | 0.57 | 0.01 | 59.97 | 0.55 | 0.62 | *** |
| AmyL_1 | **<---** | InsR_1 | 0.22 | 0.01 | 16.80 | 0.20 | 0.20 | *** |
| AmyL_1 | **<---** | PutaL_1 | 0.16 | 0.02 | 8.86 | 0.12 | 0.12 | *** |
| AmyL_1 | **<---** | PrecnR_1 | 0.14 | 0.02 | 7.55 | 0.11 | 0.11 | *** |
| dlpfcR | **<---** | dlpfcR_1 | -0.10 | 0.01 | -14.03 | -0.10 | -0.10 | *** |
| dlpfcR | **<---** | InsR | 0.15 | 0.01 | 14.94 | 0.11 | 0.11 | *** |
| AmyL | **<---** | AmyL_1 | -0.19 | 0.01 | -16.86 | -0.19 | -0.20 | *** |
| dACC | **<---** | dACC_1 | 0.09 | 0.01 | 10.04 | 0.09 | 0.09 | *** |
| PrecnL | **<---** | S1L | 0.14 | 0.01 | 20.88 | 0.26 | 0.26 | *** |
| dACC | **<---** | S1L | 0.14 | 0.01 | 13.50 | 0.17 | 0.18 | *** |
| PutaR_1 | **<---** | PutaL_1 | 0.02 | 0.01 | 1.41 | 0.01 | 0.01 | 0.158 |
| PrecnL_1 | **<---** | CaudR_1 | 0.19 | 0.01 | 25.84 | 0.30 | 0.31 | *** |
| PutaR_1 | **<---** | S1L_1 | 0.42 | 0.01 | 48.06 | 0.48 | 0.53 | *** |
| PutaR_1 | **<---** | dlpfcR_1 | 0.30 | 0.01 | 33.84 | 0.34 | 0.36 | *** |
| dACC | **<---** | PutaR | 0.09 | 0.01 | 7.38 | 0.10 | 0.10 | *** |
| dACC | **<---** | dlpfcR | 0.03 | 0.01 | 3.23 | 0.04 | 0.04 | 0.001 |
| AmyR_1 | **<---** | AmyL_1 | 0.51 | 0.01 | 46.07 | 0.48 | 0.52 | *** |
| AmyL | **<---** | InsR | 0.24 | 0.01 | 17.69 | 0.22 | 0.22 | *** |
| AmyL | **<---** | ThalL | 0.16 | 0.01 | 11.38 | 0.14 | 0.14 | *** |
| AmyR_1 | **<---** | InsR_1 | 0.19 | 0.01 | 15.70 | 0.16 | 0.17 | *** |
| PrecnL_1 | **<---** | InsL_1 | 0.12 | 0.01 | 18.49 | 0.21 | 0.22 | *** |
| PrecnL | **<---** | CaudR | 0.17 | 0.01 | 21.26 | 0.26 | 0.27 | *** |
| AmyL | **<---** | PrecnR_1 | 0.19 | 0.02 | 10.49 | 0.14 | 0.14 | *** |
| AmyL | **<---** | PutaL_1 | 0.15 | 0.02 | 8.31 | 0.11 | 0.11 | *** |
| dACC | **<---** | ThalR | 0.35 | 0.01 | 49.10 | 0.50 | 0.55 | *** |
| PrecnR | **<---** | PrecnR_1 | -0.18 | 0.01 | -15.80 | -0.18 | -0.18 | *** |
| NaccL | **<---** | InsR | 0.63 | 0.01 | 64.26 | 0.61 | 0.72 | *** |
| NaccL_1 | **<---** | InsR_1 | 0.62 | 0.01 | 62.72 | 0.60 | 0.70 | *** |
| dlpfcL_1 | **<---** | ThalR_1 | 0.23 | 0.01 | 27.79 | 0.34 | 0.35 | *** |
| dlpfcL | **<---** | ThalR | 0.21 | 0.01 | 25.18 | 0.31 | 0.32 | *** |
| AmyR | **<---** | AmyL | 0.49 | 0.01 | 45.49 | 0.46 | 0.50 | *** |
| NaccR | **<---** | PrecnL | 0.34 | 0.01 | 34.95 | 0.38 | 0.40 | *** |
| NaccR_1 | **<---** | PrecnL_1 | 0.36 | 0.01 | 36.84 | 0.40 | 0.42 | *** |
| dlpfcL_1 | **<---** | dACC_1 | 0.26 | 0.01 | 21.31 | 0.26 | 0.27 | *** |
| PrecnR | **<---** | PutaL | 0.47 | 0.01 | 43.56 | 0.46 | 0.50 | *** |
| AmyR | **<---** | AmyR_1 | -0.19 | 0.01 | -19.47 | -0.19 | -0.19 | *** |
| NaccL_1 | **<---** | CaudR_1 | 0.11 | 0.01 | 13.10 | 0.12 | 0.12 | *** |
| NaccL_1 | **<---** | AmyR_1 | 0.11 | 0.01 | 14.47 | 0.13 | 0.13 | *** |
| NaccR_1 | **<---** | S1R_1 | 0.07 | 0.01 | 9.79 | 0.13 | 0.13 | *** |
| NaccR_1 | **<---** | ThalL_1 | 0.07 | 0.01 | 8.06 | 0.10 | 0.10 | *** |
| NaccR_1 | **<---** | CaudR_1 | 0.07 | 0.01 | 8.86 | 0.11 | 0.11 | *** |
| dlpfcL | **<---** | InsL | 0.06 | 0.01 | 6.63 | 0.08 | 0.08 | *** |
| dlpfcL | **<---** | S1L | 0.05 | 0.01 | 5.12 | 0.07 | 0.07 | *** |
| dlpfcL | **<---** | PutaR | 0.09 | 0.01 | 7.64 | 0.10 | 0.10 | *** |
| AmyR | **<---** | InsR | 0.20 | 0.01 | 17.07 | 0.17 | 0.18 | *** |
| dlpfcL_1 | **<---** | PutaR_1 | 0.16 | 0.01 | 18.47 | 0.19 | 0.19 | *** |
| NaccR | **<---** | S1R | 0.07 | 0.01 | 8.81 | 0.12 | 0.12 | *** |
| NaccR | **<---** | CaudR | 0.05 | 0.01 | 6.84 | 0.09 | 0.09 | *** |
| NaccR | **<---** | ThalL | 0.06 | 0.01 | 6.62 | 0.08 | 0.08 | *** |
| NaccR | **<---** | PutaR | 0.06 | 0.01 | 9.84 | 0.11 | 0.11 | *** |
| PrecnR | **<---** | ThalL | 0.14 | 0.01 | 13.37 | 0.15 | 0.15 | *** |
| PrecnR | **<---** | S1R | 0.08 | 0.01 | 9.60 | 0.12 | 0.12 | *** |
| PrecnR | **<---** | CaudR | 0.03 | 0.01 | 3.46 | 0.04 | 0.04 | *** |
| dlpfcL | **<---** | dACC | 0.24 | 0.01 | 19.41 | 0.24 | 0.25 | *** |
| NaccL | **<---** | S1R | 0.09 | 0.01 | 9.92 | 0.10 | 0.10 | *** |
| NaccL | **<---** | ThalL | 0.11 | 0.01 | 9.14 | 0.10 | 0.10 | *** |
| PrecnR | **<---** | PutaL_1 | 0.18 | 0.01 | 16.10 | 0.18 | 0.18 | *** |
| PrecnR_1 | **<---** | PutaL_1 | -0.74 | 0.07 | -11.32 | -0.75 | -0.96 | *** |
| PutaL_1 | **<---** | PrecnR_1 | 0.85 | 0.03 | 30.47 | 0.85 | 1.25 | *** |

|  |  |  | **Patient Group - All Conditions** | | | | | |  |  |  |
| --- | --- | --- | --- | --- | --- | --- | --- | --- | --- | --- | --- |
| Path | | | *B* | *SE B* | C.R. | **β** | Z' | P | Z-test | sig. | q |
| CaudR_1 | **<---** | InsR_1 | 0.64 | 0.01 | 78.73 | 0.69 | 0.84 | *** | 34.34 | * | 0.40 |
| CaudL_1 | **<---** | CaudR_1 | 0.97 | 0.01 | 85.27 | 0.70 | 0.87 | *** | 7.54 | * | 0.10 |
| CaudR | **<---** | CaudR_1 | -0.17 | 0.01 | -14.40 | -0.17 | -0.17 | *** | 0.43 |  | 0.00 |
| CaudL_1 | **<---** | InsR_1 | 0.29 | 0.01 | 27.03 | 0.22 | 0.23 | *** | 1.57 |  | 0.01 |
| CaudL | **<---** | CaudR | 1.16 | 0.01 | 134.95 | 0.84 | 1.22 | *** | 26.19 | * | 0.37 |
| CaudL | **<---** | CaudL_1 | -0.10 | 0.01 | -15.88 | -0.10 | -0.10 | *** | 2.96 |  | -0.01 |
| S1R | **<---** | CaudL | 0.75 | 0.01 | 65.81 | 0.75 | 0.97 | *** | 48.71 | * | 0.59 |
| S1R_1 | **<---** | CaudL_1 | 0.75 | 0.01 | 65.80 | 0.75 | 0.97 | *** | 48.52 | * | 0.58 |
| InsR | **<---** | CaudL | 0.54 | 0.01 | 82.44 | 0.70 | 0.88 | *** | 30.53 | * | 0.38 |
| S1R_1 | **<---** | CaudR_1 | 0.19 | 0.02 | 12.11 | 0.14 | 0.14 | *** | -12.49 | * | -0.06 |
| S1R | **<---** | CaudR | 0.19 | 0.02 | 12.09 | 0.14 | 0.14 | *** | -12.49 | * | -0.06 |
| ThalL | **<---** | InsR | 0.34 | 0.01 | 45.75 | 0.39 | 0.41 | *** | 5.92 | * | 0.05 |
| ThalR_1 | **<---** | CaudR_1 | 0.41 | 0.02 | 21.00 | 0.35 | 0.37 | *** | -3.87 |  | -0.03 |
| ThalL | **<---** | InsR_1 | 0.08 | 0.01 | 13.22 | 0.09 | 0.09 | *** | 1.51 |  | 0.00 |
| ThalL | **<---** | S1R | 0.37 | 0.01 | 64.60 | 0.55 | 0.61 | *** | 13.36 | * | 0.14 |
| ThalL_1 | **<---** | S1R_1 | 0.38 | 0.01 | 63.61 | 0.55 | 0.62 | *** | 12.99 | * | 0.14 |
| ThalL_1 | **<---** | InsR_1 | 0.32 | 0.01 | 42.41 | 0.37 | 0.38 | *** | 4.90 | * | 0.04 |
| ThalR_1 | **<---** | CaudL_1 | 0.31 | 0.01 | 21.51 | 0.36 | 0.38 | *** | 8.16 | * | 0.06 |
| PutaL | **<---** | ThalL | 0.25 | 0.01 | 29.74 | 0.33 | 0.34 | *** | 6.93 | * | 0.05 |
| PutaL | **<---** | InsR | 0.23 | 0.01 | 32.65 | 0.35 | 0.36 | *** | 11.70 | * | 0.08 |
| InsL_1 | **<---** | ThalL_1 | 0.28 | 0.01 | 24.57 | 0.26 | 0.27 | *** | 0.98 |  | 0.01 |
| InsL_1 | **<---** | ThalR_1 | 0.07 | 0.01 | 8.79 | 0.08 | 0.08 | *** | -17.91 | * | -0.08 |
| InsL_1 | **<---** | ThalL | 0.01 | 0.01 | 1.45 | 0.01 | 0.01 | 0.146 | -0.50 |  | 0.00 |
| PutaL | **<---** | CaudR | 0.17 | 0.01 | 23.78 | 0.23 | 0.24 | *** | 1.32 |  | 0.01 |
| InsL_1 | **<---** | InsR_1 | 0.51 | 0.01 | 51.01 | 0.54 | 0.61 | *** | 37.90 | * | 0.31 |
| PrecnR_1 | **<---** | PutaL | 0.06 | 0.01 | 6.11 | 0.07 | 0.07 | *** | -6.32 | * | -0.02 |
| PutaL_1 | **<---** | InsL_1 | 0.16 | 0.01 | 16.94 | 0.22 | 0.22 | *** | 5.45 | * | 0.02 |
| PrecnR_1 | **<---** | ThalL_1 | 0.42 | 0.02 | 26.46 | 0.58 | 0.66 | *** | 13.19 | * | 0.15 |
| PrecnR_1 | **<---** | CaudR_1 | 0.26 | 0.02 | 17.05 | 0.38 | 0.40 | *** | 14.10 | * | 0.10 |
| PrecnR_1 | **<---** | CaudL_1 | 0.03 | 0.01 | 2.98 | 0.07 | 0.07 | 0.003 | -4.71 | * | -0.01 |
| PrecnR_1 | **<---** | InsL_1 | 0.35 | 0.02 | 21.85 | 0.53 | 0.59 | *** | 15.51 | * | 0.16 |
| ThalR | **<---** | CaudR | 0.41 | 0.02 | 20.97 | 0.35 | 0.37 | *** | -3.97 |  | -0.03 |
| ThalR | **<---** | CaudL | 0.31 | 0.01 | 21.53 | 0.36 | 0.38 | *** | 8.26 | * | 0.06 |
| InsL | **<---** | PutaL | 0.72 | 0.02 | 45.77 | 0.51 | 0.57 | *** | 21.37 | * | 0.20 |
| dACC_1 | **<---** | InsR_1 | 0.01 | 0.01 | 0.67 | 0.01 | 0.01 | 0.503 | -2.08 |  | 0.00 |
| InsL | **<---** | ThalR | 0.06 | 0.01 | 8.26 | 0.07 | 0.07 | *** | -16.89 | * | -0.07 |
| dACC_1 | **<---** | ThalR_1 | 0.70 | 0.01 | 109.77 | 0.85 | 1.25 | *** | 51.35 | * | 0.70 |
| InsL | **<---** | ThalL | 0.29 | 0.01 | 24.15 | 0.27 | 0.28 | *** | 7.49 | * | 0.04 |
| dACC_1 | **<---** | InsL_1 | -0.03 | 0.01 | -3.20 | -0.03 | -0.03 | 0.001 | -16.80 | * | -0.03 |
| S1L | **<---** | InsL | 0.52 | 0.01 | 49.20 | 0.48 | 0.52 | *** | 6.35 | * | 0.06 |
| S1L | **<---** | CaudR | 0.19 | 0.02 | 13.11 | 0.18 | 0.18 | *** | 5.28 | * | 0.02 |
| S1L_1 | **<---** | ThalL_1 | 0.21 | 0.01 | 14.83 | 0.19 | 0.19 | *** | 6.39 | * | 0.02 |
| S1L_1 | **<---** | InsL_1 | 0.53 | 0.01 | 44.74 | 0.49 | 0.53 | *** | 8.54 | * | 0.09 |
| S1L_1 | **<---** | dACC_1 | 0.14 | 0.01 | 13.84 | 0.13 | 0.13 | *** | -12.25 | * | -0.06 |
| S1L_1 | **<---** | CaudL_1 | 0.08 | 0.01 | 8.12 | 0.10 | 0.10 | *** | -1.10 |  | 0.00 |
| S1L | **<---** | S1R | 0.11 | 0.01 | 10.97 | 0.14 | 0.14 | *** | -1.96 |  | -0.01 |
| S1L | **<---** | ThalR | 0.11 | 0.01 | 10.95 | 0.11 | 0.11 | *** | -7.52 | * | -0.03 |
| dlpfcR_1 | **<---** | InsL_1 | 0.71 | 0.01 | 74.46 | 0.70 | 0.87 | *** | 10.12 | * | 0.14 |
| PutaR | **<---** | S1L | 0.40 | 0.01 | 44.09 | 0.51 | 0.56 | *** | 2.97 |  | 0.03 |
| PutaR | **<---** | InsL | 0.07 | 0.01 | 7.20 | 0.08 | 0.08 | *** | -18.63 | * | -0.08 |
| PutaR | **<---** | CaudR | 0.10 | 0.01 | 10.77 | 0.12 | 0.12 | *** | 5.38 | * | 0.01 |
| PutaR | **<---** | ThalR | 0.16 | 0.01 | 21.56 | 0.22 | 0.22 | *** | 16.03 | * | 0.05 |
| dlpfcR_1 | **<---** | S1L_1 | 0.17 | 0.01 | 19.23 | 0.18 | 0.18 | *** | -2.60 |  | -0.01 |
| dlpfcR | **<---** | PutaR | 0.28 | 0.01 | 30.96 | 0.24 | 0.24 | *** | -3.21 |  | -0.02 |
| dlpfcR | **<---** | InsL | 0.49 | 0.01 | 58.89 | 0.50 | 0.54 | *** | -6.55 | * | -0.07 |
| AmyL_1 | **<---** | InsR_1 | 0.50 | 0.01 | 36.83 | 0.49 | 0.54 | *** | 28.02 | * | 0.23 |
| AmyL_1 | **<---** | PutaL_1 | 0.27 | 0.03 | 10.93 | 0.18 | 0.18 | *** | 4.86 | * | 0.02 |
| AmyL_1 | **<---** | PrecnR_1 | -0.06 | 0.03 | -2.17 | -0.04 | -0.04 | 0.03 | -11.70 | * | -0.01 |
| dlpfcR | **<---** | dlpfcR_1 | -0.04 | 0.01 | -7.41 | -0.05 | -0.05 | *** | 4.78 | * | -0.01 |
| dlpfcR | **<---** | InsR | 0.25 | 0.01 | 35.10 | 0.27 | 0.28 | *** | 13.70 | * | 0.06 |
| AmyL | **<---** | AmyL_1 | -0.24 | 0.01 | -23.00 | -0.24 | -0.25 | *** | -4.29 | * | 0.02 |
| dACC | **<---** | dACC_1 | 0.00 | 0.01 | -0.14 | 0.00 | 0.00 | 0.889 | -7.44 | * | -0.01 |
| PrecnL | **<---** | S1L | 0.30 | 0.01 | 43.40 | 0.47 | 0.51 | *** | 21.10 | * | 0.18 |
| dACC | **<---** | S1L | -0.06 | 0.01 | -6.42 | -0.07 | -0.07 | *** | -20.32 | * | -0.03 |
| PutaR_1 | **<---** | PutaL_1 | 0.01 | 0.01 | 0.69 | 0.01 | 0.01 | 0.488 | -0.42 |  | 0.00 |
| PrecnL_1 | **<---** | CaudR_1 | 0.33 | 0.01 | 41.10 | 0.48 | 0.52 | *** | 17.43 | * | 0.15 |
| PutaR_1 | **<---** | S1L_1 | 0.44 | 0.01 | 50.80 | 0.56 | 0.63 | *** | 8.22 | * | 0.09 |
| PutaR_1 | **<---** | dlpfcR_1 | 0.24 | 0.01 | 25.44 | 0.28 | 0.29 | *** | -5.55 | * | -0.04 |
| dACC | **<---** | PutaR | 0.17 | 0.01 | 13.73 | 0.15 | 0.15 | *** | 4.40 | * | 0.01 |
| dACC | **<---** | dlpfcR | -0.02 | 0.01 | -2.46 | -0.02 | -0.02 | 0.014 | -5.09 | * | 0.00 |
| AmyR_1 | **<---** | AmyL_1 | 0.76 | 0.01 | 82.60 | 0.78 | 1.05 | *** | 43.85 | * | 0.57 |
| AmyL | **<---** | InsR | 0.52 | 0.01 | 39.62 | 0.52 | 0.57 | *** | 29.31 | * | 0.25 |
| AmyL | **<---** | ThalL | 0.14 | 0.02 | 9.19 | 0.12 | 0.12 | *** | -1.69 |  | -0.01 |
| AmyR_1 | **<---** | InsR_1 | -0.01 | 0.01 | -1.48 | -0.01 | -0.01 | 0.138 | -14.96 | * | -0.03 |
| PrecnL_1 | **<---** | InsL_1 | 0.16 | 0.01 | 20.07 | 0.23 | 0.24 | *** | 1.67 |  | 0.01 |
| PrecnL | **<---** | CaudR | 0.22 | 0.01 | 28.41 | 0.31 | 0.32 | *** | 4.63 | * | 0.03 |
| AmyL | **<---** | PrecnR_1 | 0.21 | 0.02 | 8.67 | 0.13 | 0.13 | *** | -1.10 |  | 0.00 |
| AmyL | **<---** | PutaL_1 | 0.15 | 0.02 | 6.72 | 0.10 | 0.10 | *** | -0.76 |  | 0.00 |
| dACC | **<---** | ThalR | 0.66 | 0.01 | 95.79 | 0.79 | 1.08 | *** | 44.18 | * | 0.58 |
| PrecnR | **<---** | PrecnR_1 | -0.12 | 0.01 | -11.14 | -0.12 | -0.12 | *** | 4.86 |  | -0.02 |
| NaccL | **<---** | InsR | 0.82 | 0.01 | 108.75 | 0.84 | 1.20 | *** | 40.76 | * | 0.57 |
| NaccL_1 | **<---** | InsR_1 | 0.75 | 0.01 | 100.31 | 0.76 | 1.00 | *** | 24.99 | * | 0.34 |
| dlpfcL_1 | **<---** | ThalR_1 | 0.26 | 0.01 | 30.63 | 0.41 | 0.44 | *** | 7.17 | * | 0.06 |
| dlpfcL | **<---** | ThalR | 0.25 | 0.01 | 27.73 | 0.37 | 0.39 | *** | 6.04 | * | 0.05 |
| AmyR | **<---** | AmyL | 0.74 | 0.01 | 81.31 | 0.76 | 1.00 | *** | 41.35 | * | 0.53 |
| NaccR | **<---** | PrecnL | 0.49 | 0.01 | 53.87 | 0.53 | 0.58 | *** | 15.28 | * | 0.15 |
| NaccR_1 | **<---** | PrecnL_1 | 0.57 | 0.01 | 62.47 | 0.61 | 0.71 | *** | 24.10 | * | 0.27 |
| dlpfcL_1 | **<---** | dACC_1 | 0.28 | 0.01 | 27.51 | 0.36 | 0.38 | *** | 8.96 | * | 0.06 |
| PrecnR | **<---** | PutaL | 0.46 | 0.01 | 47.28 | 0.49 | 0.54 | *** | 3.02 |  | 0.03 |
| AmyR | **<---** | AmyR_1 | -0.13 | 0.01 | -16.81 | -0.13 | -0.13 | *** | 5.56 | * | -0.02 |
| NaccL_1 | **<---** | CaudR_1 | 0.12 | 0.01 | 16.31 | 0.12 | 0.12 | *** | -0.42 |  | 0.00 |
| NaccL_1 | **<---** | AmyR_1 | 0.11 | 0.01 | 19.47 | 0.11 | 0.11 | *** | -1.27 |  | 0.00 |
| NaccR_1 | **<---** | S1R_1 | 0.04 | 0.01 | 6.27 | 0.09 | 0.09 | *** | -3.21 |  | -0.01 |
| NaccR_1 | **<---** | ThalL_1 | 0.03 | 0.01 | 3.02 | 0.04 | 0.04 | 0.003 | -4.86 | * | -0.01 |
| NaccR_1 | **<---** | CaudR_1 | 0.08 | 0.01 | 8.93 | 0.12 | 0.12 | *** | 0.68 |  | 0.00 |
| dlpfcL | **<---** | InsL | 0.02 | 0.01 | 2.04 | 0.02 | 0.02 | 0.041 | -4.93 | * | -0.01 |
| dlpfcL | **<---** | S1L | 0.07 | 0.01 | 7.94 | 0.10 | 0.10 | *** | 2.18 |  | 0.00 |
| dlpfcL | **<---** | PutaR | 0.05 | 0.01 | 5.20 | 0.06 | 0.06 | *** | -3.61 |  | -0.01 |
| AmyR | **<---** | InsR | 0.00 | 0.01 | -0.37 | 0.00 | 0.00 | 0.715 | -14.90 | * | -0.03 |
| dlpfcL_1 | **<---** | PutaR_1 | 0.12 | 0.01 | 18.02 | 0.14 | 0.14 | *** | -3.85 |  | -0.01 |
| NaccR | **<---** | S1R | 0.03 | 0.01 | 3.71 | 0.05 | 0.05 | *** | -5.29 | * | -0.01 |
| NaccR | **<---** | CaudR | 0.04 | 0.01 | 4.75 | 0.06 | 0.06 | *** | -2.18 |  | 0.00 |
| NaccR | **<---** | ThalL | 0.00 | 0.01 | -0.48 | -0.01 | -0.01 | 0.633 | -7.01 |  | -0.01 |
| NaccR | **<---** | PutaR | 0.20 | 0.01 | 26.77 | 0.27 | 0.28 | *** | 13.87 | * | 0.06 |
| PrecnR | **<---** | ThalL | 0.16 | 0.01 | 18.24 | 0.22 | 0.22 | *** | 5.61 | * | 0.02 |
| PrecnR | **<---** | S1R | 0.04 | 0.01 | 6.69 | 0.09 | 0.09 | *** | -2.53 |  | -0.01 |
| PrecnR | **<---** | CaudR | 0.09 | 0.01 | 11.62 | 0.13 | 0.13 | *** | 7.56 |  | 0.02 |
| dlpfcL | **<---** | dACC | 0.29 | 0.01 | 28.46 | 0.37 | 0.38 | *** | 11.50 | * | 0.08 |
| NaccL | **<---** | S1R | -0.04 | 0.01 | -6.36 | -0.05 | -0.05 | *** | -12.78 | * | -0.01 |
| NaccL | **<---** | ThalL | 0.14 | 0.01 | 13.40 | 0.13 | 0.13 | *** | 2.53 |  | 0.01 |
| PrecnR | **<---** | PutaL_1 | 0.12 | 0.01 | 11.84 | 0.13 | 0.13 | *** | -4.44 | * | -0.02 |
| PrecnR_1 | **<---** | PutaL_1 | -0.66 | 0.04 | -15.15 | -0.70 | -0.88 | *** | 7.39 | * | -0.10 |
| PutaL_1 | **<---** | PrecnR_1 | 0.844 | 0.018 | 47.996 | 0.791 | 1.07 | *** | -14.28 | * | -0.21 |

Notes. *B* = unstandardized beta weight; *SE B* = standard error of unstandardized beta weight; *p* = probability; β = standardized beta weight; *Z'* = Fisher's Z transformation of standardized beta weights; q = Cohen's q effect size index (Z'r_1_ - Z'r_2_), small = .10 - .29; medium = .30 - .49; large > .50. Z test statistic based on Fisher's z transformation. n.s. = not significant. * = significant at p < .05; ** = significant at p < .01; *** = significant at p < .001. Medium and large Cohen's q effects are highlighted by shaded rows. Tests of significance are adjusted using the Bonferroni correction. Z-test is calculated by (Z'rpatients - Z'rcontrols), where r = the standardized beta weight for a path.
