# Supplementary material for: Network Alterations in Comorbid Chronic Pain and Opioid Addiction: An Exploratory Approach
Source: Front Hum Neurosci. 2019 May 29;13:174. doi: 10.3389/fnhum.2019.00174 (PMC6548857; doi:10.3389/fnhum.2019.00174)
Supplement: Supplementary file 2 [file Table_2.DOCX]

**Supplementary Table 2.** **Connection statistics pain condition.**

|  |  |  | **Control Group - Pain Condition** | | | | | |
| --- | --- | --- | --- | --- | --- | --- | --- | --- |
|  |  |  | *B* | *SE B* | C.R. | **β** | Z' | p |
| CaudR_1 | <--- | InsR_1 | 0.48 | 0.03 | 15.52 | 0.42 | 0.44 | *** |
| CaudL_1 | <--- | CaudR_1 | 0.87 | 0.03 | 33.05 | 0.67 | 0.82 | *** |
| CaudR | <--- | CaudR_1 | -0.07 | 0.03 | -2.28 | -0.07 | -0.07 | 0.02 |
| CaudL_1 | <--- | InsR_1 | 0.29 | 0.03 | 9.63 | 0.20 | 0.20 | *** |
| CaudL | <--- | CaudR | 0.99 | 0.03 | 39.20 | 0.75 | 0.97 | *** |
| CaudL | <--- | CaudL_1 | -0.12 | 0.02 | -6.21 | -0.12 | -0.12 | *** |
| S1R | <--- | CaudL | 0.31 | 0.03 | 10.74 | 0.37 | 0.39 | *** |
| S1R_1 | <--- | CaudL_1 | 0.31 | 0.03 | 11.00 | 0.38 | 0.40 | *** |
| InsR | <--- | CaudL | 0.32 | 0.02 | 18.03 | 0.47 | 0.51 | *** |
| S1R_1 | <--- | CaudR_1 | 0.31 | 0.04 | 8.26 | 0.29 | 0.30 | *** |
| S1R | <--- | CaudR | 0.34 | 0.04 | 9.10 | 0.31 | 0.32 | *** |
| ThalL | <--- | InsR | 0.35 | 0.02 | 15.28 | 0.37 | 0.39 | *** |
| ThalR_1 | <--- | CaudR_1 | 0.59 | 0.05 | 12.60 | 0.43 | 0.46 | *** |
| ThalL | <--- | InsR_1 | 0.04 | 0.02 | 1.76 | 0.04 | 0.04 | 0.08 |
| ThalL | <--- | S1R | 0.30 | 0.02 | 16.06 | 0.39 | 0.41 | *** |
| ThalL_1 | <--- | S1R_1 | 0.32 | 0.02 | 16.52 | 0.40 | 0.43 | *** |
| ThalL_1 | <--- | InsR_1 | 0.34 | 0.02 | 14.59 | 0.36 | 0.37 | *** |
| ThalR_1 | <--- | CaudL_1 | 0.26 | 0.04 | 7.32 | 0.25 | 0.26 | *** |
| PutaL | <--- | ThalL | 0.23 | 0.03 | 9.20 | 0.26 | 0.27 | *** |
| PutaL | <--- | InsR | 0.22 | 0.02 | 9.53 | 0.27 | 0.28 | *** |
| InsL_1 | <--- | ThalL_1 | 0.37 | 0.04 | 9.87 | 0.28 | 0.29 | *** |
| InsL_1 | <--- | ThalR_1 | 0.21 | 0.02 | 10.07 | 0.26 | 0.27 | *** |
| InsL_1 | <--- | ThalL | 0.05 | 0.03 | 1.39 | 0.03 | 0.03 | 0.17 |
| PutaL | <--- | CaudR | 0.14 | 0.02 | 7.19 | 0.19 | 0.19 | *** |
| InsL_1 | <--- | InsR_1 | 0.23 | 0.04 | 6.32 | 0.18 | 0.18 | *** |
| PrecnR_1 | <--- | PutaL | 0.08 | 0.03 | 2.33 | 0.08 | 0.08 | 0.02 |
| PutaL_1 | <--- | InsL_1 | 0.14 | 0.02 | 5.82 | 0.20 | 0.20 | *** |
| PrecnR_1 | <--- | ThalL_1 | 0.40 | 0.04 | 9.56 | 0.46 | 0.49 | *** |
| PrecnR_1 | <--- | CaudR_1 | 0.17 | 0.04 | 4.32 | 0.22 | 0.23 | *** |
| PrecnR_1 | <--- | CaudL_1 | 0.07 | 0.03 | 2.49 | 0.13 | 0.13 | 0.01 |
| PrecnR_1 | <--- | InsL_1 | 0.27 | 0.04 | 7.21 | 0.41 | 0.43 | *** |
| ThalR | <--- | CaudR | 0.55 | 0.05 | 11.68 | 0.41 | 0.44 | *** |
| ThalR | <--- | CaudL | 0.26 | 0.04 | 7.21 | 0.25 | 0.26 | *** |
| InsL | <--- | PutaL | 0.52 | 0.04 | 12.80 | 0.35 | 0.36 | *** |
| dACC_1 | <--- | InsR_1 | 0.07 | 0.02 | 2.72 | 0.06 | 0.06 | 0.01 |
| InsL | <--- | ThalR | 0.21 | 0.02 | 10.09 | 0.25 | 0.26 | *** |
| dACC_1 | <--- | ThalR_1 | 0.44 | 0.02 | 28.33 | 0.64 | 0.76 | *** |
| InsL | <--- | ThalL | 0.24 | 0.04 | 6.78 | 0.19 | 0.19 | *** |
| dACC_1 | <--- | InsL_1 | 0.10 | 0.02 | 5.04 | 0.12 | 0.12 | *** |
| S1L | <--- | InsL | 0.44 | 0.02 | 17.99 | 0.41 | 0.43 | *** |
| S1L | <--- | CaudR | 0.13 | 0.04 | 3.78 | 0.11 | 0.11 | *** |
| S1L_1 | <--- | ThalL_1 | 0.15 | 0.03 | 4.35 | 0.10 | 0.10 | *** |
| S1L_1 | <--- | InsL_1 | 0.42 | 0.03 | 16.23 | 0.39 | 0.42 | *** |
| S1L_1 | <--- | dACC_1 | 0.43 | 0.03 | 14.65 | 0.35 | 0.36 | *** |
| S1L_1 | <--- | CaudL_1 | 0.08 | 0.02 | 3.75 | 0.09 | 0.09 | *** |
| S1L | <--- | S1R | 0.21 | 0.03 | 7.68 | 0.20 | 0.20 | *** |
| S1L | <--- | ThalR | 0.20 | 0.02 | 8.79 | 0.23 | 0.24 | *** |
| dlpfcR_1 | <--- | InsL_1 | 0.68 | 0.02 | 28.35 | 0.65 | 0.78 | *** |
| PutaR | <--- | S1L | 0.42 | 0.02 | 17.69 | 0.49 | 0.53 | *** |
| PutaR | <--- | InsL | 0.22 | 0.02 | 9.80 | 0.24 | 0.25 | *** |
| PutaR | <--- | CaudR | 0.09 | 0.03 | 3.43 | 0.09 | 0.09 | *** |
| PutaR | <--- | ThalR | 0.04 | 0.02 | 2.12 | 0.06 | 0.06 | 0.03 |
| dlpfcR_1 | <--- | S1L_1 | 0.19 | 0.02 | 8.31 | 0.19 | 0.19 | *** |
| dlpfcR | <--- | PutaR | 0.31 | 0.03 | 12.13 | 0.27 | 0.27 | *** |
| dlpfcR | <--- | InsL | 0.59 | 0.02 | 25.04 | 0.56 | 0.63 | *** |
| AmyL_1 | <--- | InsR_1 | 0.29 | 0.03 | 8.41 | 0.24 | 0.25 | *** |
| AmyL_1 | <--- | PutaL_1 | 0.17 | 0.05 | 3.52 | 0.12 | 0.12 | *** |
| AmyL_1 | <--- | PrecnR_1 | 0.25 | 0.05 | 5.25 | 0.18 | 0.18 | *** |
| dlpfcR | <--- | dlpfcR_1 | -0.09 | 0.02 | -5.07 | -0.09 | -0.09 | *** |
| dlpfcR | <--- | InsR | 0.16 | 0.02 | 6.77 | 0.13 | 0.13 | *** |
| AmyL | <--- | AmyL_1 | -0.17 | 0.03 | -5.98 | -0.17 | -0.17 | *** |
| dACC | <--- | dACC_1 | 0.07 | 0.02 | 3.51 | 0.07 | 0.07 | *** |
| PrecnL | <--- | S1L | 0.16 | 0.02 | 8.96 | 0.27 | 0.28 | *** |
| dACC | <--- | S1L | 0.16 | 0.02 | 6.48 | 0.20 | 0.20 | *** |
| PutaR_1 | <--- | PutaL_1 | -0.03 | 0.03 | -0.98 | -0.02 | -0.02 | 0.33 |
| PrecnL_1 | <--- | CaudR_1 | 0.25 | 0.02 | 13.32 | 0.38 | 0.40 | *** |
| PutaR_1 | <--- | S1L_1 | 0.46 | 0.02 | 22.40 | 0.54 | 0.60 | *** |
| PutaR_1 | <--- | dlpfcR_1 | 0.29 | 0.02 | 13.62 | 0.33 | 0.34 | *** |
| dACC | <--- | PutaR | 0.09 | 0.03 | 2.97 | 0.09 | 0.09 | 0.00 |
| dACC | <--- | dlpfcR | 0.01 | 0.02 | 0.56 | 0.02 | 0.02 | 0.58 |
| AmyR_1 | <--- | AmyL_1 | 0.53 | 0.03 | 21.52 | 0.53 | 0.60 | *** |
| AmyL | <--- | InsR | 0.33 | 0.04 | 9.23 | 0.28 | 0.29 | *** |
| AmyL | <--- | ThalL | 0.19 | 0.04 | 4.99 | 0.15 | 0.15 | *** |
| AmyR_1 | <--- | InsR_1 | 0.19 | 0.03 | 6.66 | 0.17 | 0.17 | *** |
| PrecnL_1 | <--- | InsL_1 | 0.08 | 0.02 | 4.77 | 0.13 | 0.13 | *** |
| PrecnL | <--- | CaudR | 0.17 | 0.02 | 8.47 | 0.26 | 0.26 | *** |
| AmyL | <--- | PrecnR_1 | 0.23 | 0.05 | 4.89 | 0.16 | 0.16 | *** |
| AmyL | <--- | PutaL_1 | 0.16 | 0.05 | 3.59 | 0.12 | 0.12 | *** |
| dACC | <--- | ThalR | 0.37 | 0.02 | 22.24 | 0.54 | 0.60 | *** |
| PrecnR | <--- | PrecnR_1 | -0.17 | 0.03 | -6.21 | -0.17 | -0.17 | *** |
| NaccL | <--- | InsR | 0.66 | 0.02 | 28.28 | 0.64 | 0.76 | *** |
| NaccL_1 | <--- | InsR_1 | 0.62 | 0.02 | 26.62 | 0.61 | 0.71 | *** |
| dlpfcL_1 | <--- | ThalR_1 | 0.25 | 0.02 | 12.20 | 0.37 | 0.38 | *** |
| dlpfcL | <--- | ThalR | 0.23 | 0.02 | 11.32 | 0.35 | 0.37 | *** |
| AmyR | <--- | AmyL | 0.53 | 0.02 | 21.89 | 0.53 | 0.59 | *** |
| NaccR | <--- | PrecnL | 0.41 | 0.02 | 17.38 | 0.45 | 0.48 | *** |
| NaccR_1 | <--- | PrecnL_1 | 0.43 | 0.02 | 18.76 | 0.49 | 0.53 | *** |
| dlpfcL_1 | <--- | dACC_1 | 0.29 | 0.03 | 9.64 | 0.30 | 0.31 | *** |
| PrecnR | <--- | PutaL | 0.45 | 0.03 | 16.87 | 0.43 | 0.46 | *** |
| AmyR | <--- | AmyR_1 | -0.13 | 0.02 | -5.53 | -0.13 | -0.13 | *** |
| NaccL_1 | <--- | CaudR_1 | 0.09 | 0.02 | 4.78 | 0.11 | 0.11 | *** |
| NaccL_1 | <--- | AmyR_1 | 0.15 | 0.02 | 8.03 | 0.17 | 0.17 | *** |
| NaccR_1 | <--- | S1R_1 | 0.07 | 0.02 | 3.92 | 0.12 | 0.12 | *** |
| NaccR_1 | <--- | ThalL_1 | 0.05 | 0.02 | 2.37 | 0.07 | 0.07 | 0.02 |
| NaccR_1 | <--- | CaudR_1 | 0.06 | 0.02 | 3.07 | 0.09 | 0.09 | 0.00 |
| dlpfcL | <--- | InsL | 0.07 | 0.02 | 2.90 | 0.08 | 0.08 | 0.00 |
| dlpfcL | <--- | S1L | 0.04 | 0.03 | 1.67 | 0.06 | 0.06 | 0.10 |
| dlpfcL | <--- | PutaR | 0.03 | 0.03 | 1.18 | 0.04 | 0.04 | 0.24 |
| AmyR | <--- | InsR | 0.21 | 0.03 | 7.39 | 0.18 | 0.18 | *** |
| dlpfcL_1 | <--- | PutaR_1 | 0.14 | 0.02 | 6.57 | 0.15 | 0.16 | *** |
| NaccR | <--- | S1R | 0.06 | 0.02 | 3.42 | 0.11 | 0.11 | *** |
| NaccR | <--- | CaudR | 0.06 | 0.02 | 3.10 | 0.10 | 0.10 | 0.00 |
| NaccR | <--- | ThalL | 0.03 | 0.02 | 1.74 | 0.05 | 0.05 | 0.08 |
| NaccR | <--- | PutaR | 0.06 | 0.02 | 3.85 | 0.10 | 0.10 | *** |
| PrecnR | <--- | ThalL | 0.16 | 0.02 | 6.60 | 0.18 | 0.18 | *** |
| PrecnR | <--- | S1R | 0.09 | 0.02 | 4.30 | 0.13 | 0.13 | *** |
| PrecnR | <--- | CaudR | 0.07 | 0.02 | 3.15 | 0.09 | 0.09 | 0.00 |
| dlpfcL | <--- | dACC | 0.27 | 0.03 | 8.80 | 0.28 | 0.29 | *** |
| NaccL | <--- | S1R | 0.08 | 0.02 | 3.93 | 0.09 | 0.09 | *** |
| NaccL | <--- | ThalL | 0.12 | 0.03 | 4.49 | 0.11 | 0.11 | *** |
| PrecnR | <--- | PutaL_1 | 0.16 | 0.03 | 6.02 | 0.16 | 0.16 | *** |
| PrecnR_1 | <--- | PutaL_1 | -0.61 | 0.12 | -4.96 | -0.61 | -0.70 | *** |
| PutaL_1 | <--- | PrecnR_1 | 0.76 | 0.06 | 12.68 | 0.77 | 1.01 | *** |

|  |  |  | **Patient Group - Pain Condition** | | | | | |  |  |  |
| --- | --- | --- | --- | --- | --- | --- | --- | --- | --- | --- | --- |
|  |  |  | *B* | *SE B* | C.R. | **β** | Z' | p | Z-test | sig. | q |
| CaudR_1 | <--- | InsR_1 | 0.71 | 0.02 | 32.68 | 0.69 | 0.86 | *** | 13.99 | * | 0.41 |
| CaudL_1 | <--- | CaudR_1 | 1.00 | 0.03 | 35.56 | 0.71 | 0.89 | *** | 2.55 |  | 0.09 |
| CaudR | <--- | CaudR_1 | -0.07 | 0.03 | -2.32 | -0.07 | -0.07 | 0.02 | -0.03 |  | 0.00 |
| CaudL_1 | <--- | InsR_1 | 0.30 | 0.03 | 10.50 | 0.21 | 0.21 | *** | 0.46 |  | 0.01 |
| CaudL | <--- | CaudR | 1.18 | 0.02 | 56.61 | 0.86 | 1.29 | *** | 10.76 | * | 0.38 |
| CaudL | <--- | CaudL_1 | -0.01 | 0.02 | -0.52 | -0.01 | -0.01 | 0.601 | 3.75 |  | -0.01 |
| S1R | <--- | CaudL | 0.62 | 0.03 | 21.23 | 0.64 | 0.76 | *** | 12.59 | * | 0.34 |
| S1R_1 | <--- | CaudL_1 | 0.68 | 0.03 | 23.96 | 0.69 | 0.85 | *** | 15.15 | * | 0.43 |
| InsR | <--- | CaudL | 0.47 | 0.02 | 32.26 | 0.69 | 0.85 | *** | 11.43 | * | 0.34 |
| S1R_1 | <--- | CaudR_1 | 0.27 | 0.04 | 6.73 | 0.19 | 0.20 | *** | -3.39 |  | -0.05 |
| S1R | <--- | CaudR | 0.31 | 0.04 | 7.77 | 0.24 | 0.24 | *** | -2.86 |  | -0.05 |
| ThalL | <--- | InsR | 0.26 | 0.02 | 12.29 | 0.26 | 0.27 | *** | -4.12 | * | -0.07 |
| ThalR_1 | <--- | CaudR_1 | 0.24 | 0.05 | 5.13 | 0.22 | 0.22 | *** | -8.28 | * | -0.15 |
| ThalL | <--- | InsR_1 | 0.10 | 0.02 | 6.18 | 0.11 | 0.11 | *** | 2.28 |  | 0.01 |
| ThalL | <--- | S1R | 0.43 | 0.02 | 29.08 | 0.62 | 0.73 | *** | 10.69 | * | 0.29 |
| ThalL_1 | <--- | S1R_1 | 0.45 | 0.02 | 29.91 | 0.65 | 0.78 | *** | 12.03 | * | 0.34 |
| ThalL_1 | <--- | InsR_1 | 0.22 | 0.02 | 10.38 | 0.23 | 0.23 | *** | -4.79 | * | -0.08 |
| ThalR_1 | <--- | CaudL_1 | 0.38 | 0.03 | 11.49 | 0.49 | 0.53 | *** | 9.22 | * | 0.19 |
| PutaL | <--- | ThalL | 0.20 | 0.02 | 9.78 | 0.28 | 0.29 | *** | 0.66 |  | 0.01 |
| PutaL | <--- | InsR | 0.16 | 0.02 | 8.40 | 0.23 | 0.23 | *** | -1.48 |  | -0.02 |
| InsL_1 | <--- | ThalL_1 | 0.33 | 0.02 | 13.75 | 0.36 | 0.37 | *** | 2.91 |  | 0.05 |
| InsL_1 | <--- | ThalR_1 | 0.08 | 0.02 | 4.45 | 0.10 | 0.10 | *** | -5.66 | * | -0.06 |
| InsL_1 | <--- | ThalL | 0.02 | 0.02 | 1.19 | 0.02 | 0.02 | 0.235 | -0.37 |  | 0.00 |
| PutaL | <--- | CaudR | 0.22 | 0.02 | 11.75 | 0.33 | 0.34 | *** | 4.96 | * | 0.07 |
| InsL_1 | <--- | InsR_1 | 0.37 | 0.02 | 15.71 | 0.41 | 0.43 | *** | 8.48 | * | 0.14 |
| PrecnR_1 | <--- | PutaL | 0.14 | 0.03 | 4.90 | 0.14 | 0.14 | *** | 2.13 |  | 0.01 |
| PutaL_1 | <--- | InsL_1 | 0.16 | 0.03 | 6.34 | 0.21 | 0.21 | *** | 0.28 |  | 0.00 |
| PrecnR_1 | <--- | ThalL_1 | 0.27 | 0.03 | 8.36 | 0.41 | 0.43 | *** | -2.00 |  | -0.05 |
| PrecnR_1 | <--- | CaudR_1 | 0.36 | 0.04 | 9.12 | 0.57 | 0.64 | *** | 14.13 | * | 0.32 |
| PrecnR_1 | <--- | CaudL_1 | 0.00 | 0.03 | -0.01 | 0.00 | 0.00 | 0.995 | -4.33 | * | -0.02 |
| PrecnR_1 | <--- | InsL_1 | 0.31 | 0.04 | 8.05 | 0.44 | 0.47 | *** | 1.15 |  | 0.03 |
| ThalR | <--- | CaudR | 0.20 | 0.05 | 4.19 | 0.18 | 0.18 | *** | -8.67 | * | -0.15 |
| ThalR | <--- | CaudL | 0.42 | 0.04 | 12.12 | 0.52 | 0.57 | *** | 10.54 | * | 0.23 |
| InsL | <--- | PutaL | 0.57 | 0.03 | 16.49 | 0.43 | 0.46 | *** | 3.28 |  | 0.07 |
| dACC_1 | <--- | InsR_1 | 0.04 | 0.03 | 1.48 | 0.04 | 0.04 | 0.138 | -0.75 |  | 0.00 |
| InsL | <--- | ThalR | 0.10 | 0.02 | 5.57 | 0.13 | 0.13 | *** | -4.37 | * | -0.05 |
| dACC_1 | <--- | ThalR_1 | 0.66 | 0.02 | 36.11 | 0.78 | 1.05 | *** | 9.85 | * | 0.33 |
| InsL | <--- | ThalL | 0.29 | 0.03 | 11.44 | 0.31 | 0.32 | *** | 4.33 | * | 0.06 |
| dACC_1 | <--- | InsL_1 | -0.04 | 0.03 | -1.54 | -0.04 | -0.04 | 0.124 | -5.38 | * | -0.01 |
| S1L | <--- | InsL | 0.52 | 0.03 | 19.08 | 0.46 | 0.50 | *** | 2.22 |  | 0.05 |
| S1L | <--- | CaudR | 0.14 | 0.03 | 4.34 | 0.14 | 0.15 | *** | 1.14 |  | 0.01 |
| S1L_1 | <--- | ThalL_1 | 0.25 | 0.03 | 7.92 | 0.24 | 0.24 | *** | 4.69 | * | 0.05 |
| S1L_1 | <--- | InsL_1 | 0.51 | 0.03 | 17.06 | 0.45 | 0.49 | *** | 2.39 |  | 0.06 |
| S1L_1 | <--- | dACC_1 | 0.15 | 0.02 | 6.27 | 0.14 | 0.14 | *** | -7.53 | * | -0.11 |
| S1L_1 | <--- | CaudL_1 | 0.06 | 0.02 | 2.61 | 0.08 | 0.08 | 0.009 | -0.41 |  | 0.00 |
| S1L | <--- | S1R | 0.11 | 0.02 | 4.38 | 0.14 | 0.14 | *** | -1.92 |  | -0.02 |
| S1L | <--- | ThalR | 0.14 | 0.02 | 6.14 | 0.15 | 0.16 | *** | -2.75 |  | -0.03 |
| dlpfcR_1 | <--- | InsL_1 | 0.55 | 0.03 | 20.93 | 0.55 | 0.61 | *** | -5.64 | * | -0.17 |
| PutaR | <--- | S1L | 0.38 | 0.02 | 16.45 | 0.46 | 0.50 | *** | -1.10 |  | -0.03 |
| PutaR | <--- | InsL | 0.12 | 0.02 | 5.10 | 0.14 | 0.14 | *** | -3.77 |  | -0.04 |
| PutaR | <--- | CaudR | 0.09 | 0.02 | 4.01 | 0.11 | 0.11 | *** | 0.62 |  | 0.00 |
| PutaR | <--- | ThalR | 0.15 | 0.02 | 8.49 | 0.21 | 0.21 | *** | 5.25 |  | 0.04 |
| dlpfcR_1 | <--- | S1L_1 | 0.26 | 0.02 | 11.11 | 0.29 | 0.30 | *** | 3.60 |  | 0.05 |
| dlpfcR | <--- | PutaR | 0.35 | 0.02 | 14.45 | 0.33 | 0.34 | *** | 2.38 |  | 0.04 |
| dlpfcR | <--- | InsL | 0.38 | 0.02 | 16.39 | 0.39 | 0.41 | *** | -7.46 | * | -0.19 |
| AmyL_1 | <--- | InsR_1 | 0.39 | 0.03 | 12.81 | 0.40 | 0.42 | *** | 6.00 | * | 0.11 |
| AmyL_1 | <--- | PutaL_1 | 0.35 | 0.05 | 6.60 | 0.25 | 0.25 | *** | 4.53 | * | 0.05 |
| AmyL_1 | <--- | PrecnR_1 | -0.06 | 0.06 | -1.12 | -0.04 | -0.04 | 0.264 | -7.38 | * | -0.03 |
| dlpfcR | <--- | dlpfcR_1 | -0.02 | 0.02 | -1.47 | -0.03 | -0.03 | 0.143 | 2.14 |  | -0.01 |
| dlpfcR | <--- | InsR | 0.23 | 0.02 | 11.71 | 0.25 | 0.25 | *** | 4.15 | * | 0.05 |
| AmyL | <--- | AmyL_1 | -0.17 | 0.03 | -6.07 | -0.17 | -0.17 | *** | 0.03 |  | 0.00 |
| dACC | <--- | dACC_1 | 0.00 | 0.02 | 0.14 | 0.00 | 0.00 | 0.89 | -2.34 |  | -0.01 |
| PrecnL | <--- | S1L | 0.32 | 0.02 | 16.66 | 0.46 | 0.49 | *** | 7.28 | * | 0.15 |
| dACC | <--- | S1L | -0.06 | 0.03 | -2.30 | -0.06 | -0.06 | 0.022 | -9.01 | * | -0.04 |
| PutaR_1 | <--- | PutaL_1 | 0.08 | 0.03 | 2.78 | 0.06 | 0.06 | 0.005 | 2.78 |  | 0.00 |
| PrecnL_1 | <--- | CaudR_1 | 0.28 | 0.02 | 14.10 | 0.41 | 0.44 | *** | 1.49 |  | 0.03 |
| PutaR_1 | <--- | S1L_1 | 0.43 | 0.02 | 20.30 | 0.51 | 0.57 | *** | -0.98 |  | -0.03 |
| PutaR_1 | <--- | dlpfcR_1 | 0.29 | 0.02 | 12.69 | 0.32 | 0.33 | *** | -0.53 |  | -0.01 |
| dACC | <--- | PutaR | 0.18 | 0.04 | 5.15 | 0.16 | 0.16 | *** | 2.14 |  | 0.02 |
| dACC | <--- | dlpfcR | 0.06 | 0.03 | 2.20 | 0.06 | 0.06 | 0.028 | 1.39 |  | 0.00 |
| AmyR_1 | <--- | AmyL_1 | 0.69 | 0.03 | 27.42 | 0.69 | 0.84 | *** | 8.29 | * | 0.26 |
| AmyL | <--- | InsR | 0.42 | 0.03 | 12.87 | 0.40 | 0.43 | *** | 4.76 | * | 0.09 |
| AmyL | <--- | ThalL | 0.16 | 0.03 | 4.86 | 0.15 | 0.15 | *** | 0.07 |  | 0.00 |
| AmyR_1 | <--- | InsR_1 | 0.01 | 0.03 | 0.35 | 0.01 | 0.01 | 0.726 | -5.34 | * | -0.03 |
| PrecnL_1 | <--- | InsL_1 | 0.19 | 0.02 | 8.61 | 0.25 | 0.26 | *** | 4.16 | * | 0.05 |
| PrecnL | <--- | CaudR | 0.20 | 0.02 | 10.38 | 0.29 | 0.29 | *** | 0.99 |  | 0.02 |
| AmyL | <--- | PrecnR_1 | 0.26 | 0.05 | 4.91 | 0.17 | 0.17 | *** | 0.45 |  | 0.00 |
| AmyL | <--- | PutaL_1 | 0.05 | 0.05 | 1.02 | 0.04 | 0.04 | 0.307 | -2.70 |  | -0.01 |
| dACC | <--- | ThalR | 0.58 | 0.02 | 30.65 | 0.70 | 0.86 | *** | 8.98 | * | 0.28 |
| PrecnR | <--- | PrecnR_1 | -0.08 | 0.03 | -3.16 | -0.09 | -0.09 | 0.002 | 2.79 |  | -0.02 |
| NaccL | <--- | InsR | 0.78 | 0.02 | 38.58 | 0.79 | 1.07 | *** | 10.41 | * | 0.35 |
| NaccL_1 | <--- | InsR_1 | 0.65 | 0.02 | 33.15 | 0.68 | 0.82 | *** | 3.71 |  | 0.12 |
| dlpfcL_1 | <--- | ThalR_1 | 0.26 | 0.02 | 11.83 | 0.38 | 0.40 | *** | 0.35 |  | 0.01 |
| dlpfcL | <--- | ThalR | 0.24 | 0.02 | 10.99 | 0.35 | 0.36 | *** | -0.08 |  | 0.00 |
| AmyR | <--- | AmyL | 0.68 | 0.03 | 27.72 | 0.67 | 0.82 | *** | 7.57 |  | 0.23 |
| NaccR | <--- | PrecnL | 0.27 | 0.03 | 10.67 | 0.30 | 0.31 | *** | -5.74 | * | -0.12 |
| NaccR_1 | <--- | PrecnL_1 | 0.35 | 0.03 | 14.03 | 0.38 | 0.40 | *** | -4.31 | * | -0.10 |
| dlpfcL_1 | <--- | dACC_1 | 0.28 | 0.03 | 11.38 | 0.35 | 0.37 | *** | 2.01 |  | 0.04 |
| PrecnR | <--- | PutaL | 0.40 | 0.03 | 15.87 | 0.42 | 0.45 | *** | -0.54 |  | -0.01 |
| AmyR | <--- | AmyR_1 | -0.09 | 0.02 | -4.45 | -0.09 | -0.09 | *** | 1.13 |  | -0.01 |
| NaccL_1 | <--- | CaudR_1 | 0.16 | 0.02 | 8.76 | 0.17 | 0.17 | *** | 2.35 |  | 0.02 |
| NaccL_1 | <--- | AmyR_1 | 0.16 | 0.02 | 10.33 | 0.16 | 0.16 | *** | -0.42 |  | 0.00 |
| NaccR_1 | <--- | S1R_1 | 0.11 | 0.02 | 5.29 | 0.24 | 0.24 | *** | 4.03 | * | 0.04 |
| NaccR_1 | <--- | ThalL_1 | 0.06 | 0.03 | 2.34 | 0.09 | 0.09 | 0.019 | 0.78 |  | 0.00 |
| NaccR_1 | <--- | CaudR_1 | 0.04 | 0.02 | 1.65 | 0.06 | 0.06 | 0.099 | -1.02 |  | 0.00 |
| dlpfcL | <--- | InsL | 0.05 | 0.02 | 2.23 | 0.06 | 0.06 | 0.025 | -0.78 |  | 0.00 |
| dlpfcL | <--- | S1L | 0.12 | 0.02 | 4.73 | 0.15 | 0.15 | *** | 3.09 |  | 0.02 |
| dlpfcL | <--- | PutaR | 0.00 | 0.03 | 0.08 | 0.00 | 0.00 | 0.939 | -1.22 |  | 0.00 |
| AmyR | <--- | InsR | 0.04 | 0.03 | 1.53 | 0.04 | 0.04 | 0.126 | -4.91 | * | -0.03 |
| dlpfcL_1 | <--- | PutaR_1 | 0.13 | 0.02 | 6.93 | 0.15 | 0.15 | *** | -0.24 |  | 0.00 |
| NaccR | <--- | S1R | 0.09 | 0.02 | 4.24 | 0.19 | 0.19 | *** | 2.81 |  | 0.02 |
| NaccR | <--- | CaudR | 0.02 | 0.02 | 0.96 | 0.04 | 0.04 | 0.338 | -2.01 |  | -0.01 |
| NaccR | <--- | ThalL | 0.03 | 0.02 | 1.16 | 0.04 | 0.04 | 0.246 | -0.17 |  | 0.00 |
| NaccR | <--- | PutaR | 0.17 | 0.02 | 7.37 | 0.22 | 0.22 | *** | 3.97 |  | 0.04 |
| PrecnR | <--- | ThalL | 0.14 | 0.02 | 6.58 | 0.21 | 0.21 | *** | 0.92 |  | 0.01 |
| PrecnR | <--- | S1R | 0.05 | 0.02 | 3.05 | 0.11 | 0.11 | 0.002 | -0.48 |  | 0.00 |
| PrecnR | <--- | CaudR | 0.09 | 0.02 | 4.40 | 0.14 | 0.14 | *** | 1.82 |  | 0.01 |
| dlpfcL | <--- | dACC | 0.28 | 0.03 | 11.30 | 0.34 | 0.36 | *** | 2.44 |  | 0.04 |
| NaccL | <--- | S1R | -0.02 | 0.02 | -0.97 | -0.02 | -0.02 | 0.333 | -3.87 |  | -0.01 |
| NaccL | <--- | ThalL | 0.11 | 0.03 | 4.18 | 0.11 | 0.11 | *** | -0.14 |  | 0.00 |
| PrecnR | <--- | PutaL_1 | 0.12 | 0.03 | 4.72 | 0.13 | 0.13 | *** | -1.18 |  | -0.01 |
| PrecnR_1 | <--- | PutaL_1 | -0.63 | 0.10 | -6.23 | -0.66 | -0.79 | *** | -3.00 |  | 0.10 |
| PutaL_1 | <--- | PrecnR_1 | 0.82 | 0.05 | 17.58 | 0.79 | 1.06 | *** | 1.71 |  | 0.06 |

Notes. *B* = unstandardized beta weight; *SE B* = standard error of unstandardized beta weight; *p* = probability; β = standardized beta weight; *Z'* = Fisher's Z transformation of standardized beta weights; q = Cohen's q effect size index (Z'r_1_ - Z'r_2_), small = .10 - .29; medium = .30 - .49; large > .50. Z test statistic based on Fisher's z transformation. n.s. = not significant. * = significant at p < .05; ** = significant at p < .01; *** = significant at p < .001. Medium and large Cohen's q effects are highlighted by shaded rows. Tests of significance are adjusted using the Bonferroni correction. Z-test is calculated by (Z'rpatients - Z'rcontrols), where r = the standardized beta weight for a path.
